# IHUP: An Integrated High-Throughput Universal Phenotyping Software Platform to Accelerate Unmanned-Aerial-Vehicle-Based Field Plant Phenotypic Data Extraction and Analysis

**DOI:** 10.34133/plantphenomics.0164

**Published:** 2024-05-15

**Authors:** Botao Wang, Chenghai Yang, Jian Zhang, Yunhao You, Hongming Wang, Wanneng Yang

**Affiliations:** ^1^ Macro Agriculture Research Institute, College of Resources and Environment, Huazhong Agricultural University, 1 Shizishan Street, Wuhan, Hubei 430070, China.; ^2^ Key Laboratory of Farmland Conservation in the Middle and Lower Reaches of the Ministry of Agriculture, Wuhan, Hubei 430070, China.; ^3^ Aerial Application Technology Research Unit, USDA-Agricultural Research Service, College Station, TX 77845, USA.; ^4^ College of Plant Science and Technology, Huazhong Agricultural University, Wuhan, Hubei 430070, China.

## Abstract

With the threshold for crop growth data collection having been markedly decreased by sensor miniaturization and cost reduction, unmanned aerial vehicle (UAV)-based low-altitude remote sensing has shown remarkable advantages in field phenotyping experiments. However, the requirement of interdisciplinary knowledge and the complexity of the workflow have seriously hindered researchers from extracting plot-level phenotypic data from multisource and multitemporal UAV images. To address these challenges, we developed the Integrated High-Throughput Universal Phenotyping (IHUP) software as a data producer and study accelerator that included 4 functional modules: preprocessing, data extraction, data management, and data analysis. Data extraction and analysis requiring complex and multidisciplinary knowledge were simplified through integrated and automated processing. Within a graphical user interface, users can compute image feature information, structural traits, and vegetation indices (VIs), which are indicators of morphological and biochemical traits, in an integrated and high-throughput manner. To fulfill data requirements for different crops, extraction methods such as VI calculation formulae can be customized. To demonstrate and test the composition and performance of the software, we conducted case-related rice drought phenotype monitoring experiments. In combination with a rice leaf rolling score predictive model, leaf rolling score, plant height, VIs, fresh weight, and drought weight were efficiently extracted from multiphase continuous monitoring data. Despite the significant impact of image processing during plot clipping on processing efficiency, the software can extract traits from approximately 500 plots/min in most application cases. The software offers a user-friendly graphical user interface and interfaces for customizing or integrating various feature extraction algorithms, thereby significantly reducing barriers for nonexperts. It holds the promise of significantly accelerating data production in UAV phenotyping experiments.

## Introduction

Large-scale population genetic studies that incorporate seasonal growth monitoring have proven to be highly effective in breed high-yielding and strongly stress-resistant cultivars [1]. Phenotypic characteristics are key indicators to assess the results of different genetic varieties under environmental influence for crop breeding and improvement [2]. The use of data collected from advanced sensors in breeding improvement and growth monitoring has gained significant recognition and promotion. However, the fusion of long-time series and multisource observed data remains challenging, especially when attempting to couple observations with crop physiological mechanisms. The primary challenge lies in extending the measurement, processing, and analysis of phenotypic data to interdisciplinary research teams or to nonexperts. Such processes, especially when conducted on an unmanned aerial vehicle (UAV) platform, require the utilization of multiple software packages and expertise in photogrammetry, spectral analysis, and data modeling [3].

In recent years, several sophisticated hardware platforms with multiple sensors have been designed for automated or semiautomated measurements of plant traits, known as high-throughput phenotypic platforms [4]. These platforms mostly worked indoors or in a controlled environment with proprietary software for control systems and data processing and usually had satisfactory performance for trait-related feature extraction [5]. However, limitations of terrain, high cost, and relatively fixed hardware facilities for trait investigation make it difficult to carry out investigations in different fields [6]. In addition, inefficient and subjective manual labor is still not a satisfactory solution for investigating crop traits in large populations and at large scale, which also significantly reduces the pace of crop breeding.

The advancement of multisensor and UAV technology has led to the widespread adoption of low-altitude remote sensing UAVs for acquiring and monitoring crop growth data [7]. Sensors mounted on UAVs work as a noncontact measurement platform by which phenotypic information can be nondestructively retrieved [8]. These UAV platforms are characterized by simple deployment for quick collection of high temporal and high spatial resolution images. These images enable the extraction of crucial agronomic traits during the critical growth phase, offering a vital foundation for making informed decisions regarding planting management and breeding strategies [9]. Large datasets generated by UAV flights and continuous observation are also naturally suited for training and assessing deep learning models, which are expanding the possibilities for artificial-intelligence-enabled phenotypic research [10].

Typically, in phenotyping trials, the specific set of crop traits of interest often varies depending on the crop type and the objectives of the study. Apparently, it is evident that developing software with fixed algorithms to extract specific traits from particular crops is not a universally applicable solution for reducing the complexity of phenotypic extraction from UAV images, especially for multidisciplinary teams. Furthermore, this approach will significantly raise the costs associated with hardware and software maintenance, especially concerning sensor modifications and updates to existing algorithms [9]. Apart from these concerns, high-frequency UAV flights will inevitably result in a huge amount of timing sequence data, making data production before analysis combined with development of crop mechanisms a catastrophically time-consuming and costly job. Given the aforementioned concerns, the need for a software platform with high-throughput processing capability, flexible scalability, and low technical threshold for crop-phenotypic-data-based UAV images is extremely urgent.

Substantial efforts have been devoted to simplifying and optimizing the processing flow. Chen and Zhang [11] developed a Python library with graphical user interface (GUI), called GRID, to perform plot segmentation and extraction of vegetation index (VI). Easy MPE was created to convert coordinates in raw photo images (*u*, *v*) of each plot through calibrated internal and external camera parameters [12]. FIELDimagedR and R/UAStools::plotshpcreate were also developed as plot segmentation software, with the former used to count crop plants and the latter focused on creating shapefiles [4,13]. The tools mentioned above were an attempt to automate the process of creating region of interest (ROI) shapefiles; however, the results were inaccurate when one plant overlapped another [12]. Furthermore, such software could not perform personalized data extraction and analysis because it was limited to the input image bands [11]. With a suite of original algorithms, AirMeasurer was developed to extract biologically relevant information on rice, such as crop height, canopy coverage, and growth rate [14]. Its current algorithms were developed for rice traits and cannot be generalized to other crops. HSI-PP focused on extracting and analyzing plant phenotypic traits from hyperspectral images by integrating preprocessing, feature extraction, and modeling functions [15]. Many modeling methods, such as support vector machines and random forests, were integrated and could be directly selected for data analysis. However, researchers’ own algorithms or models could not be incorporated into the complete process from data production to application.

Breeding and cultivation experiments that involve frequent monitoring of crop growth [16] impose stricter requirements on the accompanying data processing software. In particular, software usage logic needs to be designed in a simple way for nonexpert users. Besides, phenotypic data extraction from massive numbers of images obtained by continuous and simultaneous observations at multiple experimental sites has to be a highly efficient. Expansibility of software should be enhanced, and the data extraction strategy should be designed to be highly flexible, enabling users to customize trait calculation formulae or integrate different deep-learning-based algorithms [17].

Therefore, the goal of this study was to develop an Integrated High-Throughput Universal Phenotyping (IHUP) software system as an accelerator for researchers facing extremely heavy data processing workloads to extract and analyze data from phenotyping experiments. The major functionalities of the software should include (a) preprocessing functions such as ROI creation to perform phenotypic extraction from UAV-based images efficiently without using multiple software platforms; (b) processing of large volumes of temporal data in batches with all required parameters; (c) easy integration of Python scripts into the software to enable researchers’ exclusive deep-learning-based image feature extraction algorithms for phenotypic data production; and (d) a user-friendly GUI that simplifies the process of extracting data such as image features, VIs, and structural information. Consequently, IHUP should reduce the technical difficulty for extracting plot-level agronomic traits from UAV images.

## Materials and Methods

### Development of high-throughput phenotypic extraction software

**Fig. 1.**
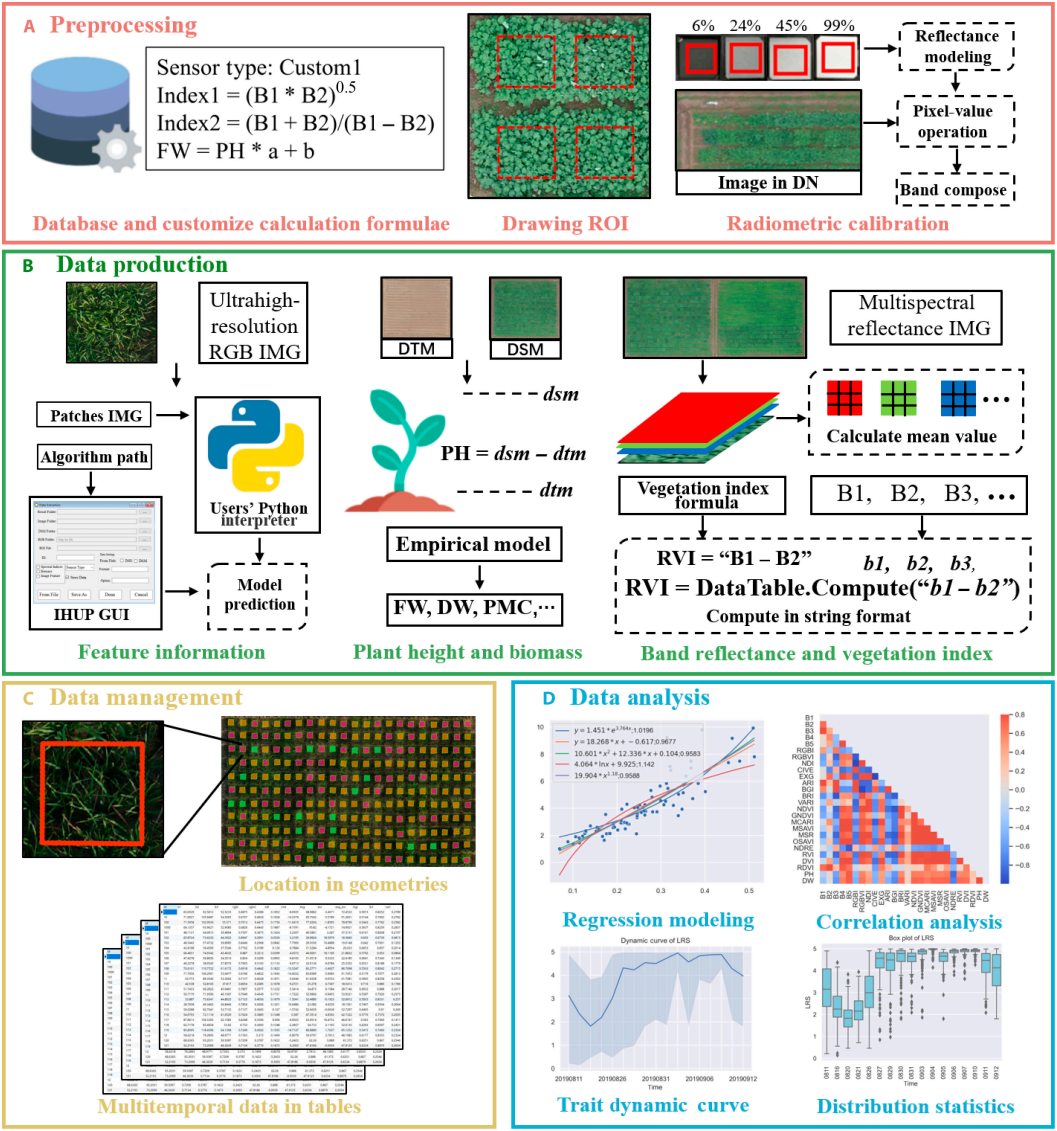
Functional modules of developed phenotyping software. (A) Preprocessing, (B) data production, (C) data management, and (D) data analysis. FW, fresh weight; DW, drought weight; PMC, plant moisture content; DN, digital number; DTM, digital terra map refers to the bare soil elevation of the field.

Figure 1 shows the process of extracting plot-level phenotypic data from multisource UAV images. The process includes 4 main modules: preprocessing, data extraction, data management, and statistical analysis.

#### Preprocessing

Field experiments consist of plots of different crop varieties or different treatments and controlled conditions. The spatial shape of the ROI for each plot needs to be determined, with reference to a map or an orthoimage. The ROIs for the plots must be clearly defined through polygon vertices stored in a shapefile. Accordingly, the preprocessing module was designed to create shapefiles and to store the label and feature attributes of each plot. The creation of ROI requires manual drawing, while the planting range of numerous plots could be determined through replication. The radiometric calibration function was used to convert pixel values of orthophotos from digital numbers to reflectance. Radiometric calibration is necessary to compare data from different imaging devices at different times and places using the empirical linear method [18]. Various red–green–blue (RGB) and multispectral (MS) sensors mounted on UAVs have different band ranges and center wavelengths [19], which may lead to a VI deriving from the bands with a discriminative physiological meaning. To process images obtained from different sensors, the customized extraction strategy function was developed to establish a tailored strategy for extracting VIs, biomass, and image features. A calculation method for phenotypic features was then defined through this function.

#### Data production

As defined by the user or by default, the extraction strategy included 3 methods to calculate or extract data from corresponding images. Specifically, spectral indices, which could be calculated through VI formula and be defined freely such as the normalized difference vegetation index. In addition, the common VI formulae for RGB or MS images were preset in the software seen in Table 1. Plant height was extracted by a digital surface map (DSM) deviation calculation, while biomass was estimated using a customized empirical formula based on prior knowledge or established from previous survey data. Image feature information was predicted using specified algorithms or a deep learning model that incorporated users’ local Python files for crop trait extraction such as rice leaf rolling score (LRS).

**Table 1.** Preset spectral index calculation formulae for RGB and MS sensor

Index	Formula	Sensor
BGI	$\begin{array}{c} b/g \end{array}$	RGB/MS
BRI	$\begin{array}{c} b/r \end{array}$	RGB/MS
CIVE	$\begin{array}{c} r*0.441-g*0.811+b*0.385+18.78745 \end{array}$	RGB/MS
EXG	$\begin{array}{c} \left(g*2-r-b)/(r+g+b\right) \end{array}$	RGB/MS
EXR	$\begin{array}{c} \left(r*1.4-g)/(r+g+b\right) \end{array}$	RGB/MS
EE	$\begin{array}{c} \mathrm{EXG}+\mathrm{EXR} \end{array}$	RGB/MS
NDI	$\begin{array}{c} \left(g-r)/(g+r\right) \end{array}$	RGB/MS
RGBI	$\begin{array}{c} r/g \end{array}$	RGB/MS
RGBVI	$\begin{array}{c} \left(g*g-b*r)/(g*g+b*r\right) \end{array}$	RGB/MS
VARI	$\begin{array}{c} \left(g-r)/(g+r-b\right) \end{array}$	RGB/MS
ARI	$\begin{array}{c} \left(1/g)-(1/\mathrm{re}\right) \end{array}$	MS
DVI	nir−r	MS
GNDVI	$\begin{array}{c} \left(\text{nir}-g)/(\text{nir}+g\right) \end{array}$	MS
MCARI1	$\begin{array}{c} 1.2*(2.5*(\text{nir}-r)-1.3*(\text{nir}-g)) \end{array}$	MS
MSAVI	$\begin{array}{c} \text{nir}*2+1-\sqrt{{\left(\text{nir}\;*\;2+1\right)}^{2}-8*(\text{nir}-r)} \end{array}$	MS
MSR	$\left(\text{nir}/r-1)/(\sqrt{\text{nir}/r}+1\right)$	MS
NDRE	(nir−re)/(nir+re)	MS
NDVI	(nir−r)/(nir+r)	MS
RDVI	$(nir-r)/\sqrt{nir+r}$	MS
OSAVI	1.16∗(nir−r)/(nir+r+0.16)	MS
RVI	nir/r	MS

#### Data management

Three data management solutions suitable for different analysis methods were integrated by the IHUP: database, shapefile, and table. Typically, the system would generate a table file (*.xlsx) and a shapefile (*.shp) to store the result data. This choice was made because the table is the one of the most commonly used data formats in data analysis and the shapefile is the most universal vector file storage format, which records geometries and associated traits. Database storage was optional; if desired, the software would create a new table to record the extracted data. The database format is more powerful for retrieving and managing millions of phenotypic traits in large-population experiments [20].

#### Data analysis

To mine knowledge from massive phenotypic data, statistical analysis functions were incorporated to examine the distribution and change of observed data as well as to screen key indicators for subsequent analysis. With surveyed ground truth data, regression modeling was developed to fit regression models between traits of interest and observed phenotypic data according to several commonly used mathematical functions (Note S1). Correlation analysis was developed to screen for the phenotypic traits with the highest correlation coefficients to the true values. A figure displaying the correlation matrix of all measured values could be generated. Trait dynamic curve was developed to show the change trend of observed data of interest, including the maximum and average of all plots in a field. By showing box plots for each phenotypic data point at each date, the distribution statistics function was designed to confirm the validity of the data.

### Case study: Rice drought phenotype monitoring

**Fig. 2.**
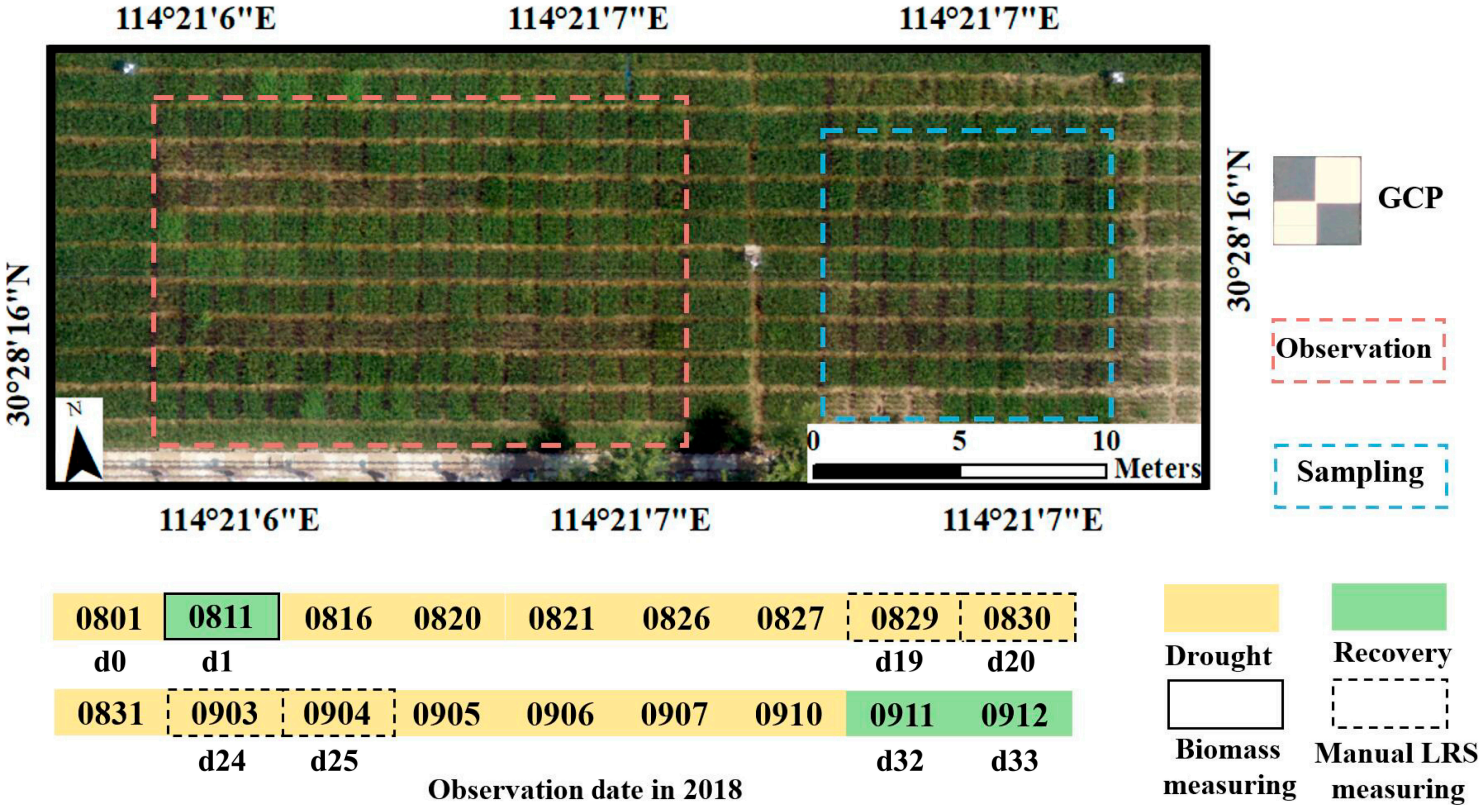
Study area for rice drought phenotype monitoring. GCP, ground control point.

The objective of this experiment was to screen for drought-resistant rice varieties through multitemporal drought characteristics derived from UAV images. Experiments were carried out at the Experimental Station (30°28′15.84″ N, 114°21′6.42″ E) of Huazhong Agricultural University in Wuhan, China. A total of 78 accessions with 3 replications (234 plots) were phenotyped and evaluated under drought stress conditions created by withholding water. Plant water content and LRS were successfully used to map drought-resistance genes in rice accession. More details can be found in our previous study [21]. The data from this earlier study were processed by the IHUP software to demonstrate its application potential in phenotyping research. The study area and sampling times are shown in Fig. 2.

During the monitoring period, a total of 18 sessions of UAV data were reprocessed, including digital orthophoto maps of RGB and MS images. The RGB images were captured using the Sony RX1R II camera mounted on the DJI Matrice 600 Pro, and the MS images were captured using the RedEdge-3 camera mounted on the DJI Phantom 4. Both RGB and MS orthoimages were generated through Metashape. DSM data were generated through captured RGB images. The ground sampling distance of RGB, DSM, and MS orthoimages was 0.27, 1.09, and 1.7 cm per pixel, respectively. Details of the UAV flight mission and the raw image information were described in a previous study [21]. Field investigation included manual measurement of fresh weight, dry weight, and LRS, which were used as sensitive indicators to reflect drought stress [22]. At day 1 (d1), the fresh weight and drought weight of 78 plots (in the sampling zone) were measured destructively. During the monitoring period, manual LRS (*LRS_m*) was measured as the average of 3 experts’ assessments (at d19 to d20 and d24 to d25), and the estimated LRS from UAV images (*LRS_e*) was predicted through a deep learning model trained in a previous study [21]. This model and prediction of *LRS_e* were integrated into IHUP, and the correlations between *LRS_m* and the VIs were calculated. Because of the lack of information regarding the bare soil height in the field, plant height was estimated by calculating the mean value within each plot, representing the elevation after subtracting the soil height. Plant height and biomass were fitted to an equation that could be used in the IHUP to calculate biomass in follow-up phenotyping experiments.

### Performance testing

For the work described in this section, the software was used to process a set of test data (totaling 40 RGB and MS images) from the case study outlined in the “Case study: Rice drought phenotype monitoring” section to illustrate its performance under various conditions according to an orthogonal test. By which, significance indicators could be determined through statistical analysis [23]. Up to 1,000 ROIs and 40 time series of data were tested, and the times of extracting VIs and LRS were recorded. In total, LRS and 21 VIs, such as normalized difference vegetation index (details in Table 1), were extracted for performance testing. Software performance was assessed on the basis of processing efficiency, measured as the number of plots processed within 1 min. Each test was repeated 3 times for accuracy and consistency. Orthogonal experimental design, analysis of variance, and significance testing were performed by the Minitab statistical software package (Minitab Version 18, Minitab Inc., State College, PA, USA). Experiments were conducted on a consumer-grade workstation (CPU: Intel Core i9-9900K @ 3.60GHz).

## Results

### Development of IHUP

#### Overview of IHUP

**Fig. 3.**
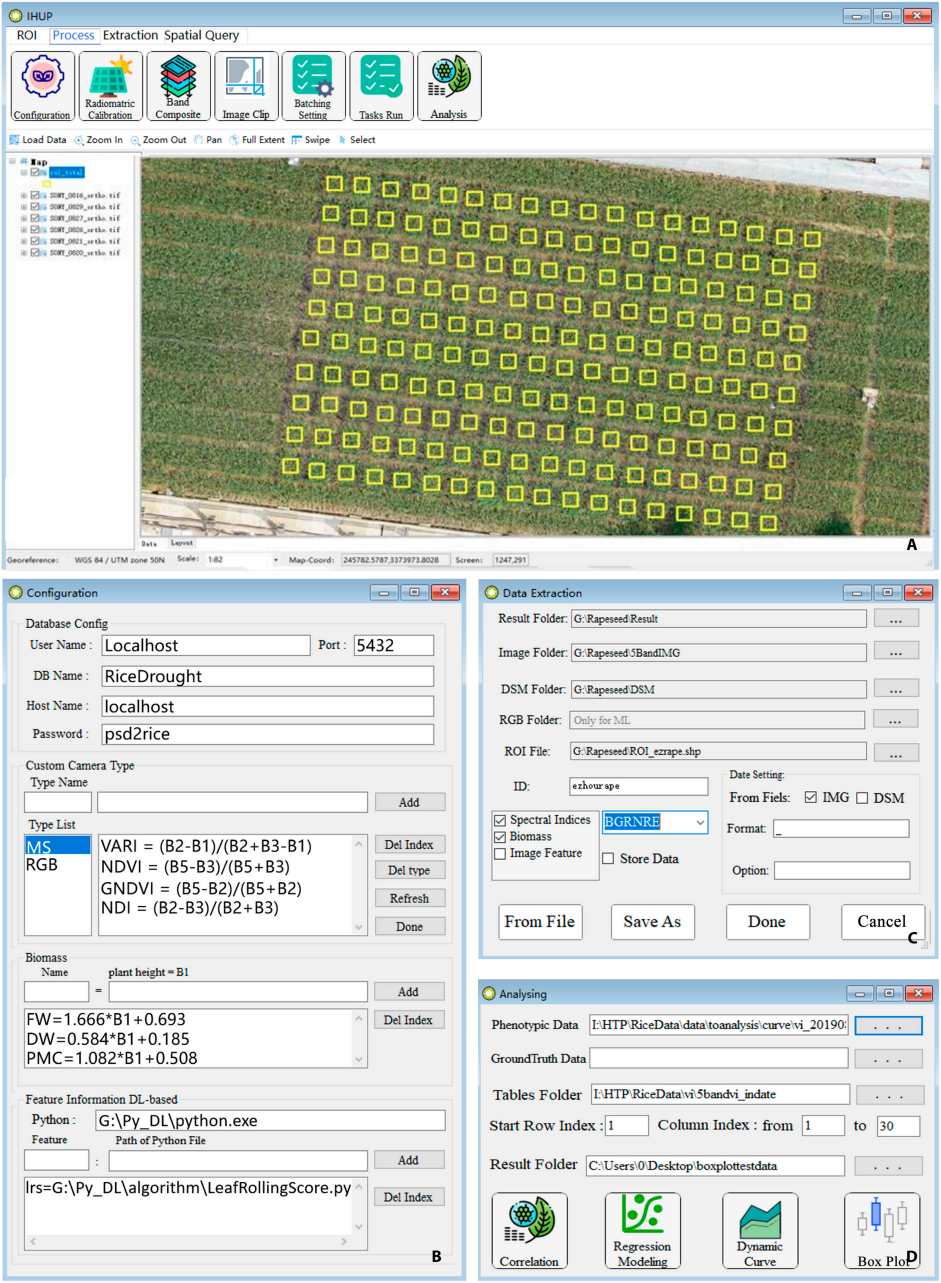
GUI of IHUP software.

IHUP is a WIN64 application with an interactive GUI developed by C# using Visual Studio 2017 and is currently supported only on Microsoft Windows. Several underlying image processing functions and algorithms provided by the Pixel Information Expert Secondary Development Kit 5.2 (PIE-SDK, http://www.piesat.cn/en/PIE-SDK.html) were used to clip patches from the digital orthophoto map and to edit the field attributes in shapefiles. The main interface of the developed software is shown in Fig. 3.

#### Data sources and processing results

IHUP was developed to extract plot-level phenotypic data from multisource UAV image data and ROI files. Tiff (*.tiff, *tif) was the default image file format supported by the software, and several field-based images acquired by various sensors (RGB, MS, hyperspectral, thermal infrared, etc.) could be processed. The software could calculate the indices of interest according to customized VI formula for specified sensor types, even though it could not identify the band composition of the input image. The software designated MS images, DSMs, and RGB images as raw data.

Within the result path, folders were generated based on flight dates, wherein output and intermediate files were stored with a task name and a time stamp. Each subdirectory contained a table and a shapefile that encompassed spatial location and phenotypic data stored within the attribute table. Plot-level image files clipped by ROIs were saved in the output folder, although there was no need for researchers to review them after extraction. After time series data processing, table files were named according to the same rule as filenames. In addition, a database could be established to record the data for each date, contingent upon users configuring the connection settings.

#### Preset spectral index calculation formulae

Two commonly used sensors with their corresponding index calculation formulae were built into the software: RGB and MS. In other words, the default input image was consisted of either 3 visible bands or 3 visible bands and near-infrared and red-edge bands. Various spectral indices were calculated on the basis of the above bands, and their applications are given in Table 1.

### Application to phenotypic acquisition for rice drought stress response

**Fig. 4.**
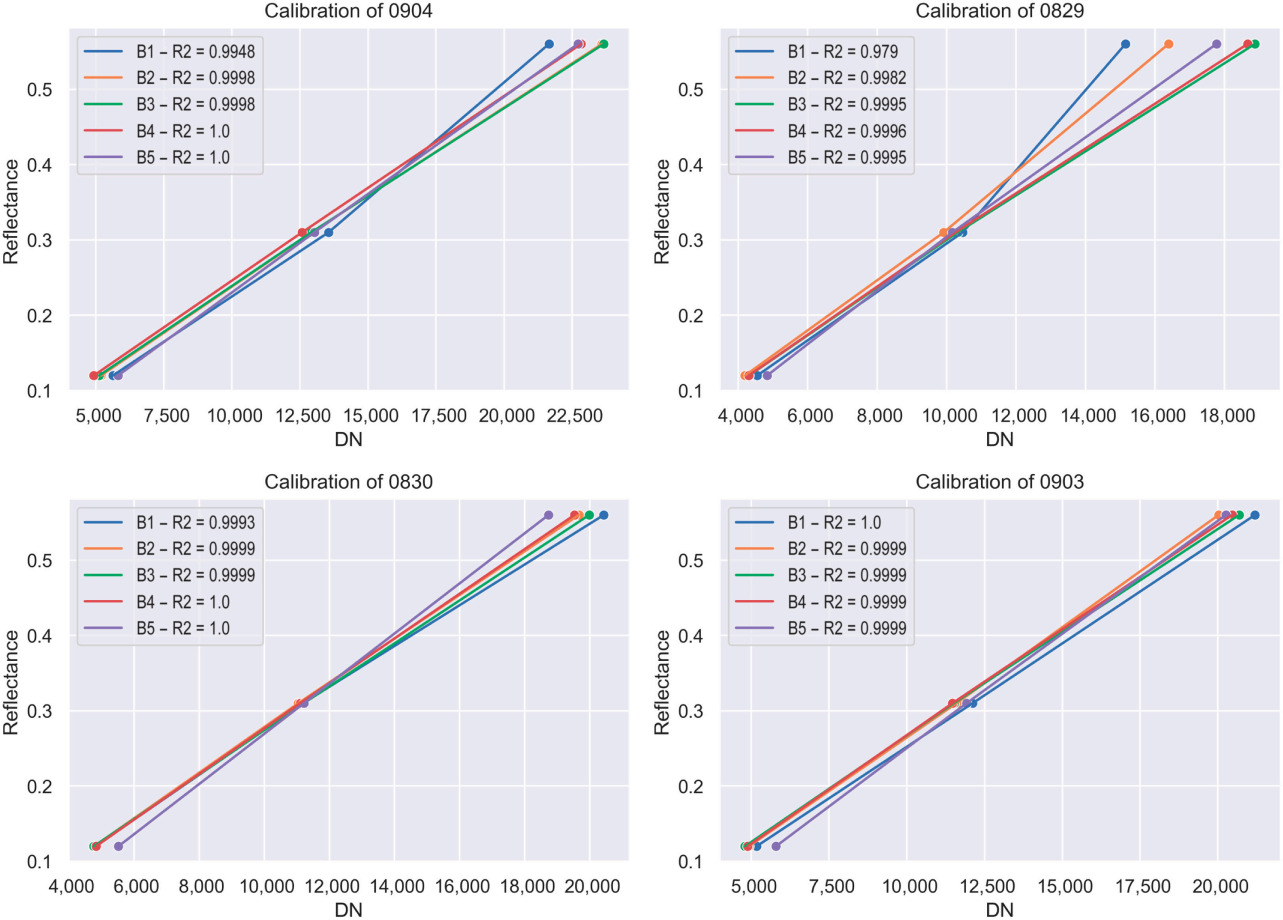
Reflectance calibration curves and accuracy of 4 test MS images.

Four MS images captured on manual LRS measurement dates were calibrated by IHUP, in which the reflectance values of the 3 calibration boards were 0.12, 0.32, and 0.56, respectively. Figure 4 shows the reflectance calibration curves and accuracy, which were directly plotted by the software. The detailed transformation equations are recorded in Table S1.

**Fig. 5.**
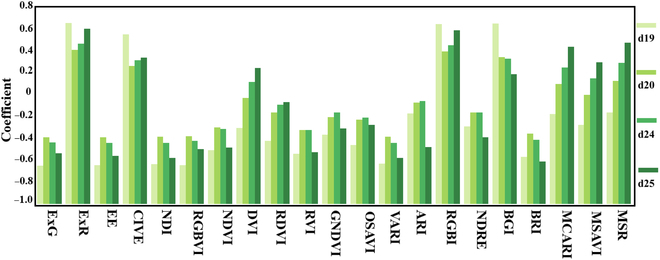
Correlation coefficients between LRS*_m* and extracted VIs on 4 dates.

A correlation analysis was conducted between *LRS_m* on 4 dates and multiple spectral indices extracted using IHUP. As shown in Fig. 5, several RGB color indices had a fairly high correlation with *LRS_m*, such as the excess-red index (ExR), the color index of vegetation, the red–green ratio index, and the pigment index. Among these, ExR and red–green ratio index showed a steady correlation trend. Calculated results would be rendered by the software as pictures in the form of a correlation matrix. The original correlation matrix is provided in Table S2.

**Fig. 6.**
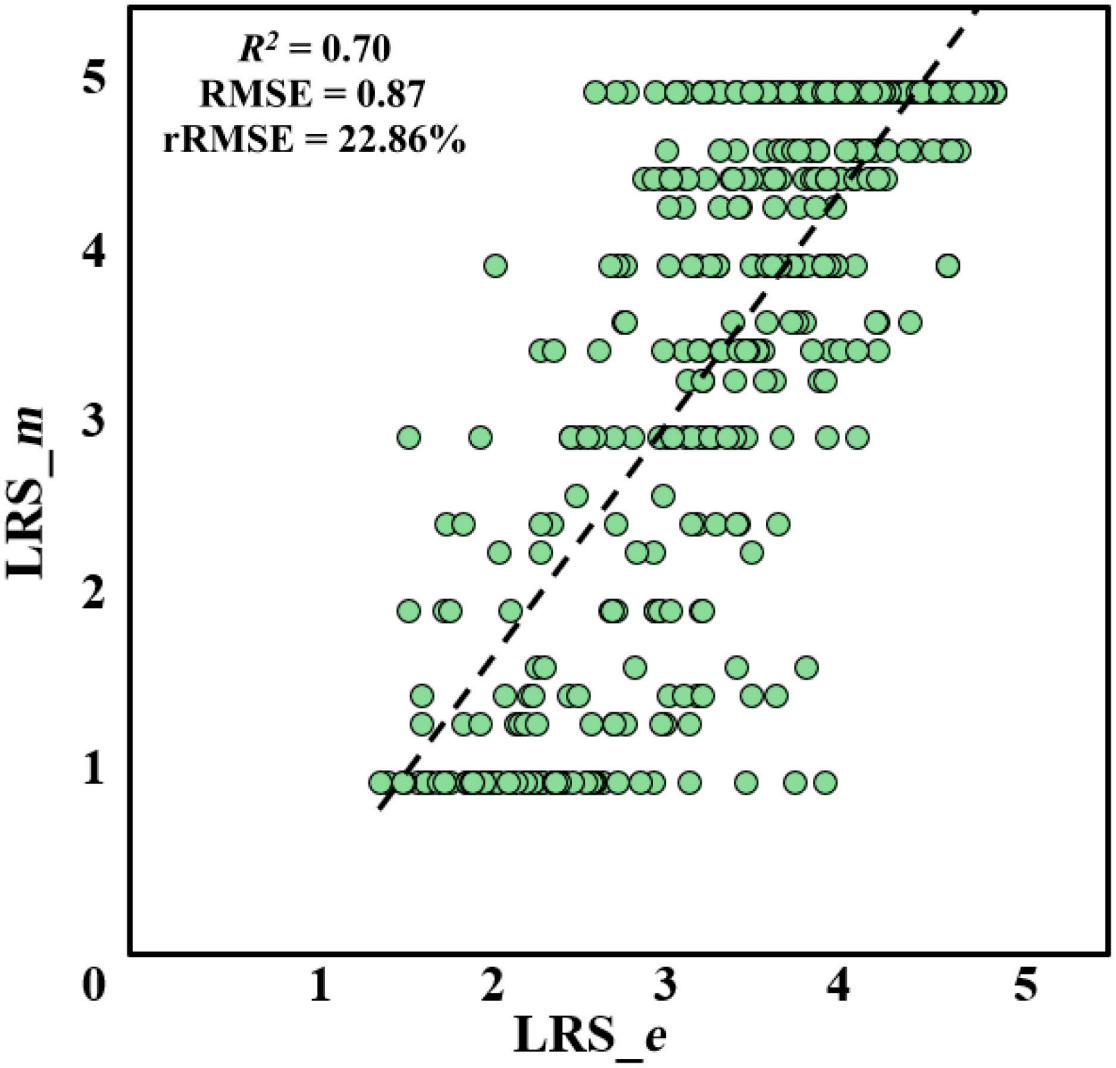
Accuracy of LRS estimated by an integrated model.

*LRS_m* was used to assess the accuracy of the previous LRS scoring model. *LRS_e* was automatically predicted as a result of integrating the model throughout the processing. As shown in Fig. 6, the predicted values were generally lower than the ground truth. The accuracy indicators of the prediction model were calculated, including *R*^2^ (0.70), root mean square error (RMSE) (0.87), and relative RMSE or rRMSE (22.86%). Table S3 gives the predicted LRS.

**Fig. 7.**
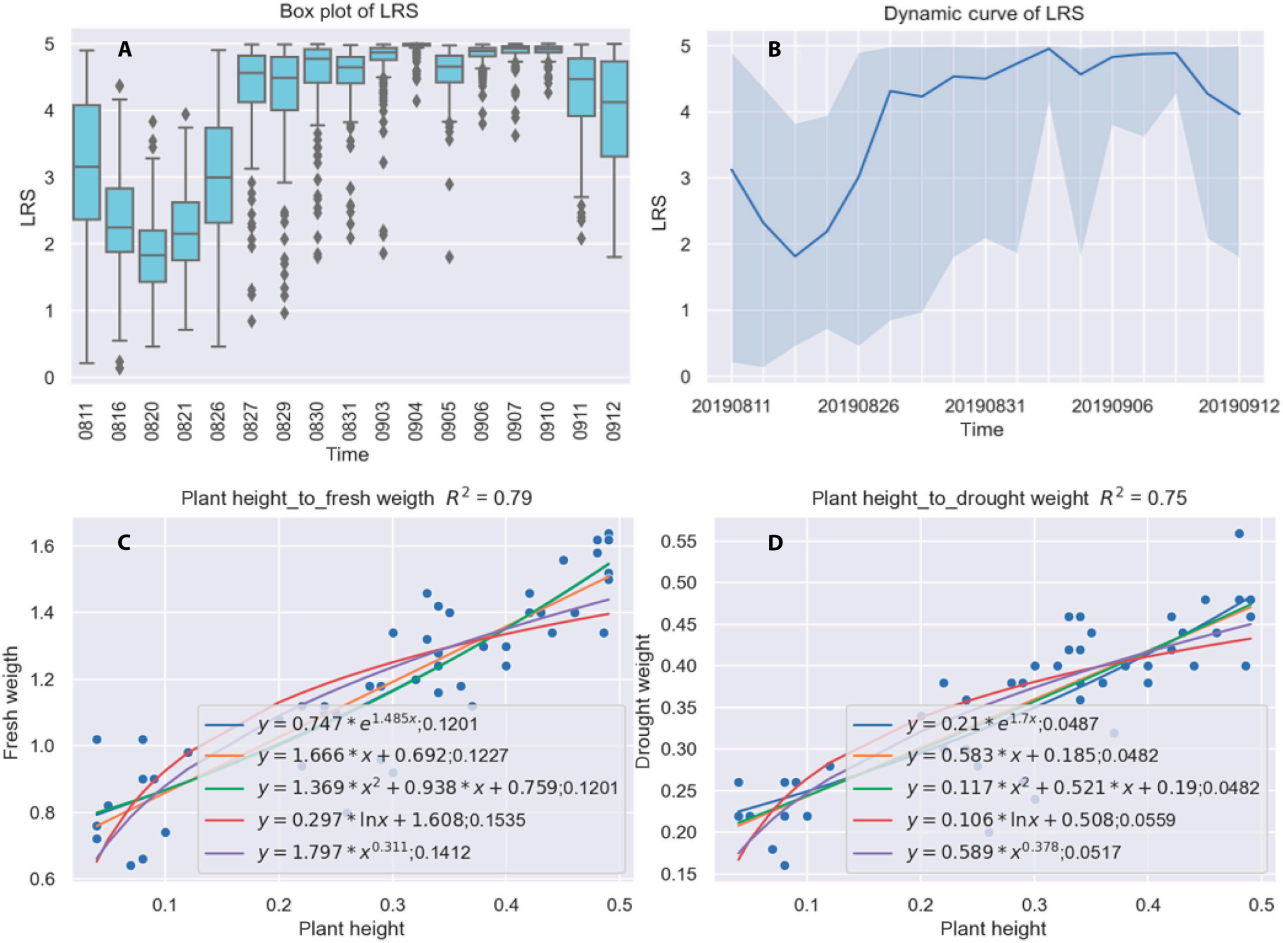
Analyzed resulting figure generated by IHUP. (A) Box plot of LRS. (B) Dynamic curve of LRS. (C) Plant height to fresh weight. (D) Plant height to drought weight.

The analysis function was used to complete the figure generation including the LRS distribution and dynamic curve shown in Fig. 7A and B. The fitted model between estimated plant height and measured biomass was plotted by IHUP. Figure 7C shows the fitted result of plant height versus fresh weight, and Fig. 7D shows the result for plant height versus drought weight. The software performed curve fitting according to any of 5 functions by default (Note S1), including linear and exponential functions. The fitted equations and their RMSE were added to the legends in resulting image, and the coefficient of determination for the best fit equation was displayed in the image title. The best coefficients of determination were 0.79 between plant height and fresh weight and 0.75 between plant height and drought weight. For fresh weight, both the exponential regression and second-degree polynomial regression had the best fit with the same RMSE of 0.1201 kg/m^2^ (Fig. 7C). For drought weight, both linear regression and second-degree polynomial regression produced the best accuracy with the same RMSE of 0.0482 kg/m^2^ (Fig. 7D). The measured weight and plant height data are shown in Table S4. Missing units and caps of scattered and fitted lines (see legends of Fig. 7C and D) might appear in pictures automatically generated by software as units are not recognized by software or tables.

In this work, the LRS prediction model and data from the case study were utilized to demonstrate the functionality of the IHUP. The goal was for the software to possess features that enable the easy extraction of crop phenotypic data from UAV orthoimage images, thereby advancing the utilization of UAV remote sensing technology in crop phenotyping research. In addition, the integration of the LRS model served to illustrate the IHUP software,s capability and ease of integrating existing algorithms. For the computation of LRS using this software, a resolution of 0.3 cm per pixel or higher is recommended.

### Software processing performance

Table 2 shows the experimental design and the software performance results. In total, 16 experiments were performed to evaluate the processing efficiency and stability of the software, and each experiment was performed 3 times. Four factors were selected, including plot, period, database, and deep learning. Plot and period refer to the number of plots and the number of test images, respectively, database represents whether the result data were stored in a database, and deep learning indicates whether image feature information (*LRS_e*) was estimated using the deep-learning-based LRS prediction model.

**Table 2.** Results of software performance test

Test	Factors				Working time (min)				Efficiency (plots/min)
	Plot	Period	DB	DL	*R*-1	*R*-2	*R*-3	*R* mean	
T1	484	10	√	√	8.3	8.19	8.25	8.25	586.90
T2	484	15	√	√	12.31	12.68	12.5	12.50	580.95
T3	484	25	×	×	7.35	7.9	6.98	7.41	**1,632.93**
T4	484	40	×	×	12.51	12.37	12.19	12.36	1,566.77
T5	600	10	√	×	4.01	4.21	4.07	**4.10**	1,464.61
T6	600	15	√	×	6.13	6.05	6.04	6.07	1,481.89
T7	600	25	×	√	25.94	25.76	25.81	25.84	580.57
T8	600	40	×	√	41.94	42.03	42.31	42.09	570.16
T9	800	10	×	√	16.02	15.45	15.1	15.52	515.35
T10	800	15	×	√	25.08	24.86	24.3	24.75	**484.91**
T11	800	25	√	×	17.66	17.09	17.45	17.40	1,149.43
T12	800	40	√	×	26.46	26.85	26.19	26.50	1,207.55
T13	1,000	10	×	×	6.7	5.98	6.65	6.44	1,551.99
T14	1,000	15	×	×	10.65	10.8	10.21	10.55	1,421.35
T15	1,000	25	√	√	43.87	43.2	44.9	43.99	568.31
T16	1,000	40	√	√	75.96	74.5	72.1	**74.19**	539.18

DB, database; DL, deep learning. The values highlighted in bold represent the longest and shortest processing times, as well as the highest and lowest processing efficiencies.

The efficiency ranged from 484.91 plots/min for T10 to 1,632.93 plots/min for T3 among the 16 tests. If computationally intensive deep learning model was used for prediction, the efficiency was much lower. T10 was the least efficient, but the phenotypic data of approximately 500 plots could still be extracted successfully from the UAV image every minute. On the other hand, if the software did not need to call the deep learning model for prediction, it could process up to 1,646 plots/min as in T3. Table 3 shows an analysis of variance of the work efficiency results. Apparently, working efficiency was affected significantly by the number of plots and whether deep learning models were calculated. This was obvious because the array loop was slowed down by an increase in the number of plots and because the deep learning models ran more slowly than the simple numerical calculations.

**Table 3.** Analysis of variance results for working efficiency factors

Source	DF	Adj SS	Adj MS	*F* value	*P* value
Plot	3	134,123	44,708	8.99	0.008
Period	3	4,501	1,500	0.30	0.824
DB	1	27,246	27,246	5.48	0.052
DL	1	3,108,760	3,108,760	625.03	0.000
Error	7	34,816	4,974		
Total	15	3,309,446			

DF, degrees of freedom; Adj SS, adjusted sum of squares of deviation; Adj MS, adjusted mean squares.

**Table 4.** Controlled trial design for software working efficiency

Trial 1				Trial 2			
Test	Plot	Index	Times	Test	Index	Plot	Times
T1-1	484	13	10	T2-1	5	800	10
T1-2	600	13	10	T2-2	10	800	10
T1-3	800	13	10	T2-3	15	800	10
T1-4	1,000	13	10	T2-4	20	800	10
T1-5	1,500	13	10	T2-5	30	800	10
T1-6	2,500	13	10	T2-6	40	800	10
T1-7	4,000	13	10	T2-7	50	800	10
T1-8	6,000	13	10	T2-8	60	800	10
T1-9	8,000	13	10	T2-9	70	800	10
T1-10	10,000	13	10	T2-10	80	800	10

## Discussion

### Performance efficiency for phenotype extraction

**Fig. 8.**
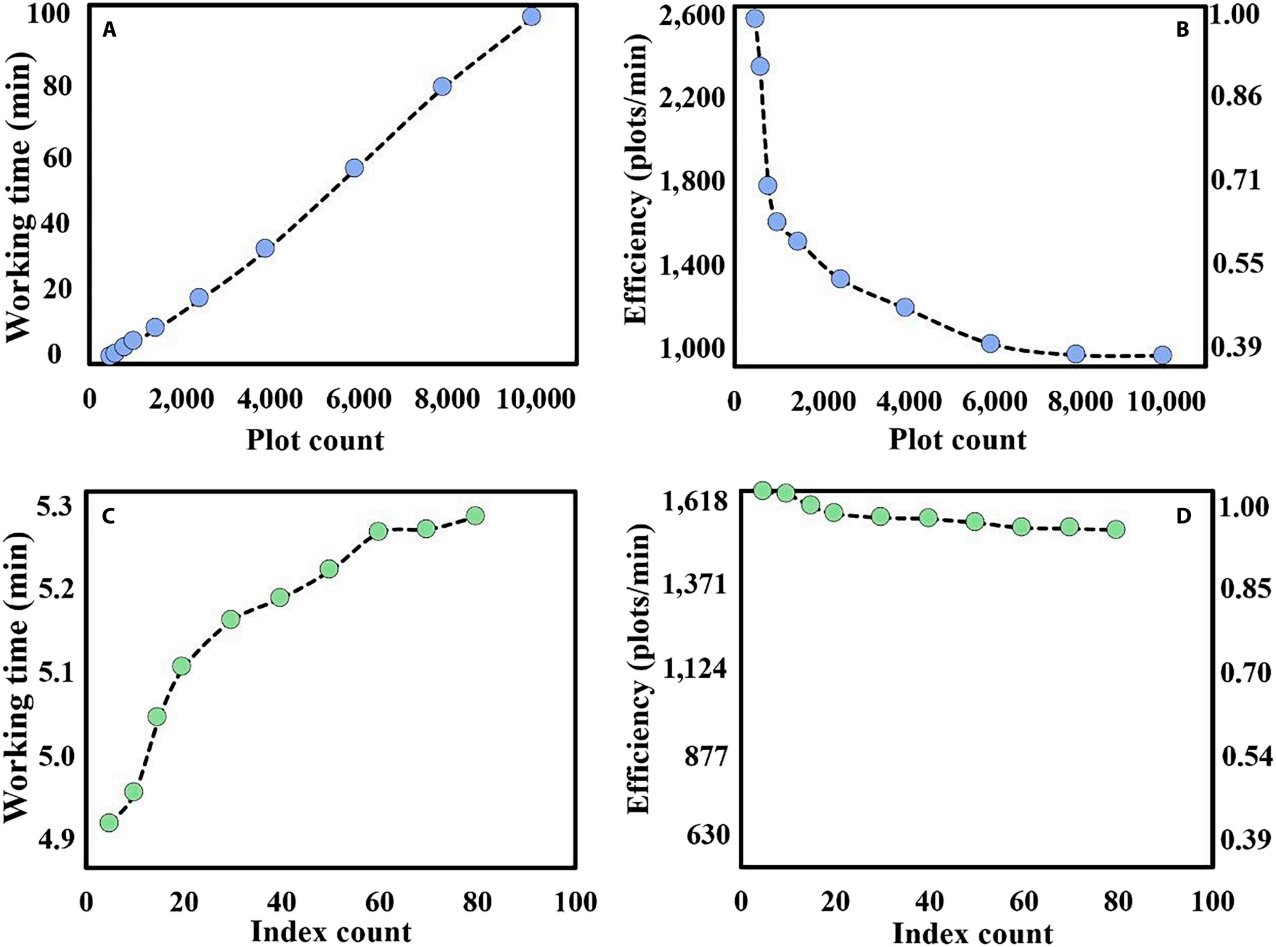
Plot and VI count impact on software performance.

Simple and efficient data extraction from UAV data was primary for field-based phenotyping experiments for nonexperts in remote sensing. The data extraction process involved initially clipping each image by ROI for each plot, followed by conducting statistical and numerical calculations, which constituted the 2 primary steps. To determine the influence of computation processing on software operation, 2 controlled trials on the number of plots and extractions of the test VIs were designed as shown in Table 4. These tests did not involve any database storage or model prediction. Detailed trial results can be found in Table S5. Trends in working time and efficiency (number of ROIs processed in 1 min) for the 2 trials are plotted in Fig. 8. The maximum working efficiencies of the 2 trials (2,547 and 1,617 plots/min, respectively) were considered as the reference and labeled as 1.00 in Fig. 8B and 8D.

As indicated by Fig. 8, working efficiency would be reduced by increasing the number of plots or the number of VIs. During processing of each plot, working time was mainly composed of clipping and other operations such as numerical calculations and assignments. Typically, image processing tasks (such as ROI clipping) took more time compared to numerical operations (VI calculations). Therefore, working efficiency would be higher when the software prioritized performing numerical operations. When faced with a substantial workload, particularly when clipping a large number of plot images, the processing efficiency primarily relied on the speed of clipping. As a result, the significant efficiency reduction trend displayed in Fig. 8B was well attributed to the fact that the bulk of the time was spent on the large amount of plot clipping. Conversely, there was no significant decrease in efficiency when the number of VI extractions increased, as depicted in Fig. 8D. The efficiency remained at approximately 94% of its highest level (1,617) when calculating 80 VIs. Although multithreading is designed to accelerate array or loop iterations, detailed image processing procedures such as reads or writes have not been optimized. This could explain the obvious decline in efficiency shown in Fig. 8B when the number of plots increased, while the number of calculated VIs remained constant.

It is important to highlight that the decision to extract plot image feature information using deep learning models had a notable impact on the software’s working efficiency, as demonstrated by the statistical results presented in Table 3. In general, invoking deep learning to extract a feature was a more time-consuming process than calculating a VI through numerical operations. Indeed, this was primarily due to the software utilizing local commands to communicate with the Python interpreter, facilitating the transmission of algorithm execution parameters and the predicted result for each image. While invoking the deep learning model led to an increase in working time, as indicated in Table 2, the most critical factor affecting software working efficiency was the image clipping process for each plot.

### High-throughput universal phenotyping software

IHUP was developed as an accelerator for UAV-platform-based crop phenotypic data production and analysis. Substantial plot-level phenotypic data were extracted from multisource UAV images and saved in tables or shapefiles, which contributed greatly not only to specific gene analysis of different crop varieties but also to the understanding of phenotypes and their relationship to their environment. As mentioned, whether for phenotypic experiments or for growth monitoring, phenotypic data must be extracted over time as a basis for population analysis or precise management decisions. However, the processing and analysis of time series and multisource data obtained from growth monitoring pose major challenges. Such difficulties will be exacerbated if additional image preprocessing is required, particularly for nonexperts in remote sensing. The software was primarily focused on resolving the data processing overload when extracting phenotypic data from UAV images.

The most commonly used cameras mounted on UAVs for crop phenotype acquisition are RGB and MS cameras. The software was configured to include spectral index formulae specific to these camera types, allowing for the easy extraction of conventional VIs by nonexperts. However, crops of various species may require specific calculation methods tailored to their agronomic traits. The ability to customize extraction methods and incorporate existing algorithms is thus a vital aspect of data production. For example, IHUP allows for the integration of estimation models for different species, including deep learning models developed by other researchers, such as PRNet, which was specifically trained for rice panicle ratio estimation [24]. Such traits as panicle ratio and LRS are usually extracted through scripts independently of the VI calculation. Moreover, those traits may be recombined into complex traits representing crop physiological and stress performance [10].

**Fig. 9.**
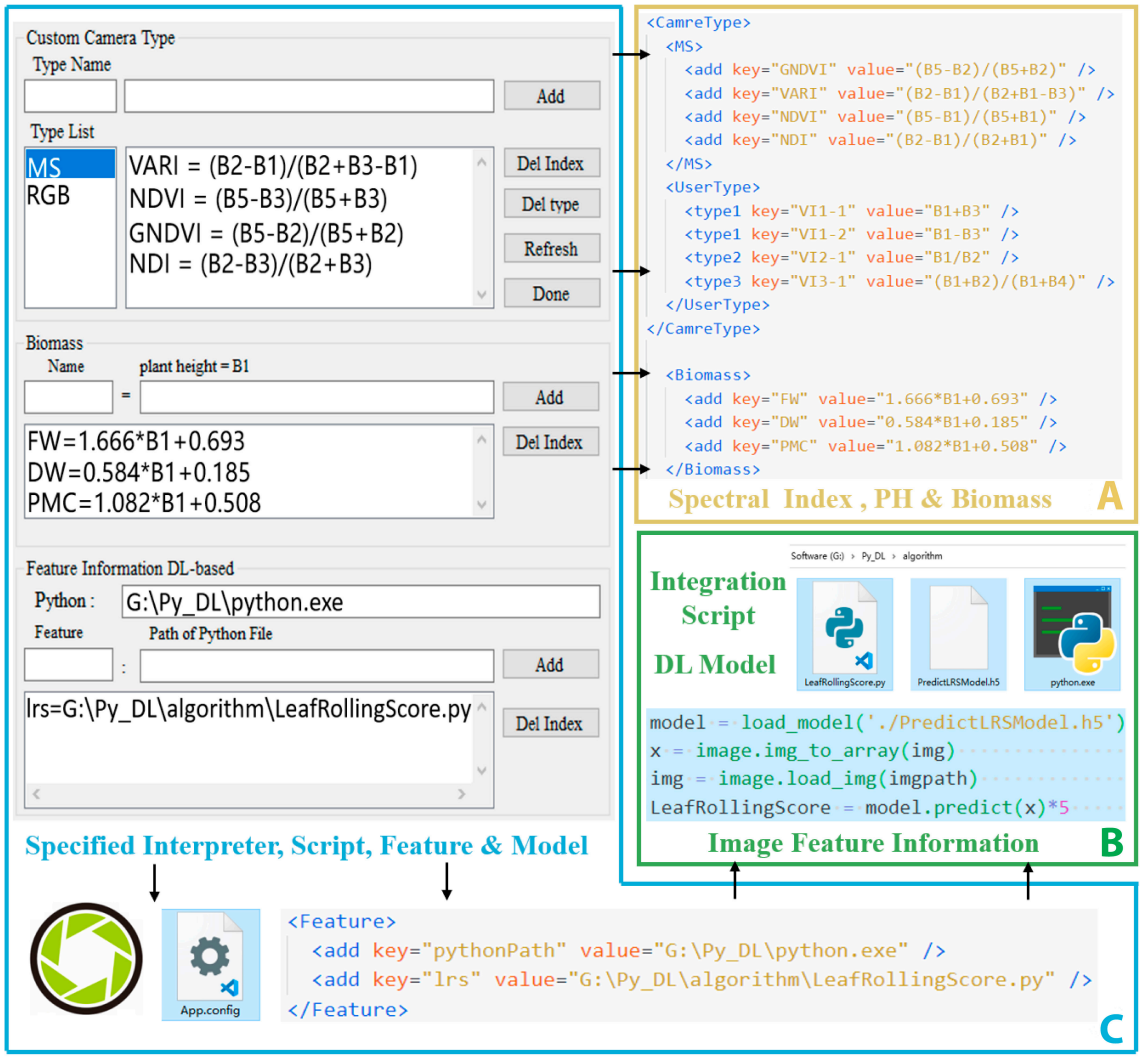
Extensibility of phenotypic data extraction strategy.

IHUP was developed with a user-friendly interface, which is illustrated in Figs. 3B and 9C, allowing users to create additional data extraction methods that can be applied to research involving diverse species. Figure 9A and B demonstrate the utilization of Python code to define the extraction strategy for VIs, biomass, and plant height. The Python code highlighted in blue was used to predict LRS using the deep learning model, as depicted in Fig. 9B. In particular, researchers can construct their own extraction strategies to extract biomass and VIs from DSMs and reflectance images. The calculation formulae are entered through the GUI as shown in Fig. 9C and are recorded locally as depicted in Fig. 9A and C. Besides, the software installation includes a simple Python runtime to illustrate the extensibility for integrating other algorithms. This runtime is only used for LRS prediction and data analysis work without the need for configuration. To personalize every aspect of the data extraction approach, researchers simply need to interactively complete the settings in Fig. 9C, which include local Python interpreter and local script. In this scenario, the software utilizes the user’s local Python environment to perform feature extraction based on the specified script provided by the user. The IHUP software outputs the extracted features. It is worth noting that IHUP automatically configures input parameters and communication methods for this script based on relevant parameters set for the computing task, such as the ROI and RGB orthoimage. A demo Python script for integrate local algorithm could be referenced in Note S2. For more detailed information, refer to the user guide provided in Note S3.

### Limitations and future improvement

The IHUP software included the 3 processing modules (i.e., preprocessing, data extraction, and data analysis) for phenotypic data extraction based on multisource UAV images. These modules were capable of conducting all the necessary data conversions from reflectance images to phenotypic data tables, with the exception of orthophoto production. IHUP primarily focused on data extraction and analysis from field orthophotos. If the research objective changes or the trait extraction algorithms are updated, data processing needs to be repeated. However, in such cases, orthophoto generation is typically not repeated, despite it being a crucial step that cannot be skipped after image acquisition.

UAV image mosaicking and orthophoto generation require sophisticated digital photogrammetry algorithms, and there are few open-source software packages that can build a user-friendly interface to implement these processes. Open-source drone mapping software, OpenDroneMap (https://github.com/OpenDroneMap/ODM), is mainly run from the command line. In addition, several commercial software packages have contributed to UAV-based phenotyping experiments, including Pix4Dmapper (https://www.pix4d.com/pix4dmapper), Metashape (https://www.agisoft.com/), and DJI Terra (https://www.dji.com/dji-terra). With support for a variety of sensor types, these software packages have been widely used in UAV-based field phenotypic research [25]. Consequently, the integration of the orthophoto mosaic function was not implemented in IHUP. However, a step-by-step user guide detailing the process of orthophoto generation with Metashape, as mentioned in Note S4, could prove to be beneficial.

Delineating accurate plot ROIs in orthophoto is important to ensure quality data extraction with the consideration of slight deviations of the images obtained at different time periods. IHUP does not incorporate plot segmentation, requiring the manual creation of plot ROIs, which can be a laborious process, particularly when dealing with a significant number of plots. Several libraries have been developed to address this issue by thresholding, photogrammetry, or image features [11–13]. Nonetheless, the effectiveness and accuracy of most of these methods are not superior to manual ROI creation, particularly in situations where the canopy is closed or nearly closed [12].

Data extraction requires a marked time investment. Clipping plots from an orthoimage and invoking a deep learning model have a significant impact on working efficiency, but these steps are indispensable. Extracting image feature information through a Python script might reduce the processing efficiency due to the predicted values that need to be recorded by IHUP through local commands. In addition, developing a program capable of directly reading ROI values without the need for clipping can potentially enhance the processing throughput of software extraction for phenotypic data.

## Data Availability

The IHUP software developed in this study is available from https://drive.google.com/uc?export=download&id=1aZalN0yqli9l2pqyQAPK0IiACs7UhrHF. For further usage details, please contact the corresponding author.

## Supplementary Materials

Supplementary 1 Notes S1 to S4 Movie S1 Tables S1 to S5 Guidelines for software testing
