# Supplementary material for: IHUP: An Integrated High-Throughput Universal Phenotyping Software Platform to Accelerate Unmanned-Aerial-Vehicle-Based Field Plant Phenotypic Data Extraction and Analysis
Source: Plant Phenomics. 2024 May 15;6:0164. doi: 10.34133/plantphenomics.0164 (PMC11335093; doi:10.34133/plantphenomics.0164)

Dear reviewers：

We apologize that the software could not be tested following the standard procedures due to an expired activation code. To rectify this issue, we have provided valid activation codes, allowing for testing of the software to improve our manuscripts. Below are some essential components that you may require for testing the software. We would like to express our sincere gratitude for your efforts and time. Should you have any further inquiries or questions during the testing process, please feel free to contact us at jz@mail.hzau.edu.cn. We truly appreciate your insightful suggestions, and we hope that these revisions will facilitate the smooth progress of the testing, ultimately leading to the successful publication of the manuscript.

**Software installation download links (IHUPInstallation.zip)：**

(Google Drive):

https://drive.google.com/uc?export=download&id=1aZalN0yqli9l2pqyQAPK0IiACs7UhrHF

(Baidu Netdisk):

https://pan.baidu.com/s/1uhZqNrRz1-mWiaTdZXtluQ?pwd=1668

**Test data download links (TestData.zip):**

(Google Drive):

https://drive.google.com/uc?export=download&id=151xpgXIFfKkBxg1EMvW7hJTiyN_ZjU_k

(Baidu Netdisk):

https://pan.baidu.com/s/1aWQLV6RA22S10L87aT_Xbw?pwd=1668

**Video download links (IHUPVideo.mp4):**

(Google Drive):

https://drive.google.com/uc?export=download&id=14GbHWXjizRRh3VoSmPYis2G99NJHuNgu

(Baidu Netdisk):

https://pan.baidu.com/s/10gUiK14uDPGnABqVCfnfug?pwd=1668

**Activation code:**

XLXF-TT7U-UFP1-MXJB, expire on February 22, 2024

**Activation steps:**

1. Download and install the VirBox via [link](https://download.virbox.com/virboxtools/V5_SS_Setup_2_4_0_50200.zip), which is used to activate IHUP.


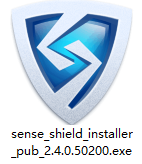


**2.** Sign up via this [link](https://e.lm.virbox.com/activity/register.html?region=CN&developerGuid=F50F35C4C74DB02F5666D2FA17372AD74958242673932E34EF853B5878A89F0F60DE17929C9E236000918AE8E3967AEA) by email.


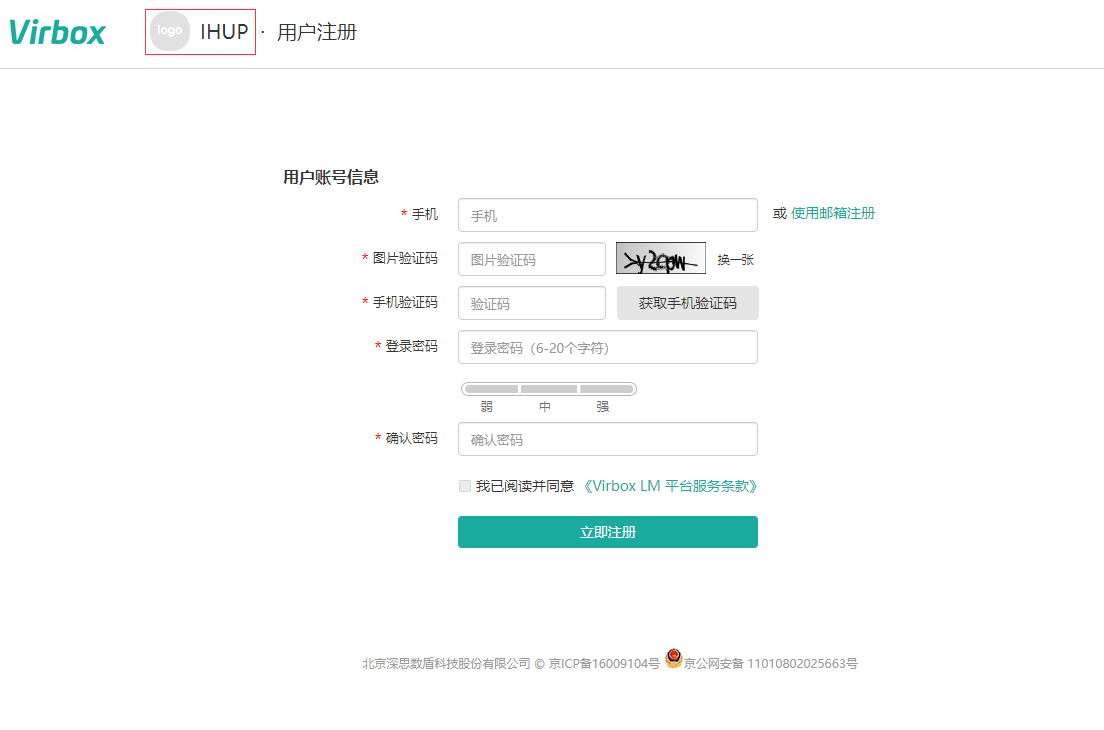


3. Log in with your account and use the activation code to activate your account.


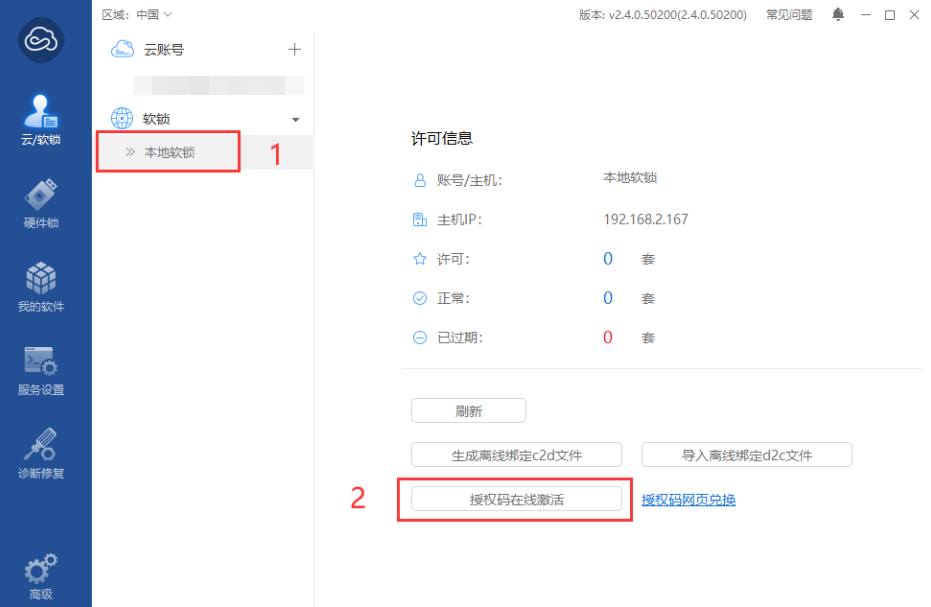


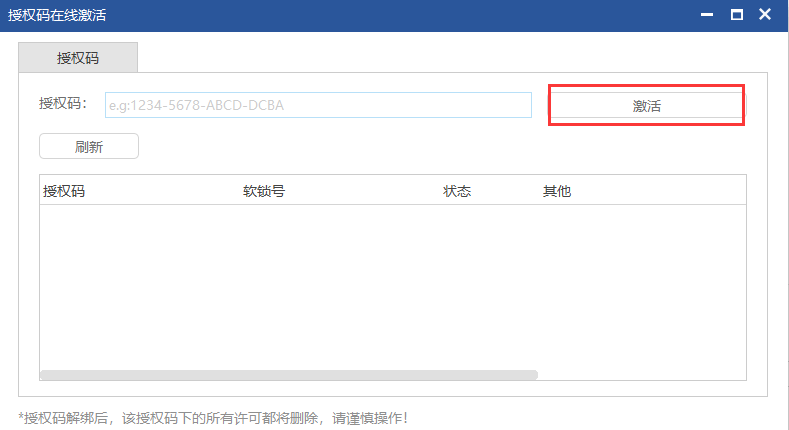

Supplement: Supplementary 1 — Notes S1 to S4 Movie S1 Tables S1 to S5 Guidelines for software testing [file plantphenomics.0164.f1.zip › Guidelines for Software Testing.docx]
