# Supplementary material for: IHUP: An Integrated High-Throughput Universal Phenotyping Software Platform to Accelerate Unmanned-Aerial-Vehicle-Based Field Plant Phenotypic Data Extraction and Analysis
Source: Plant Phenomics. 2024 May 15;6:0164. doi: 10.34133/plantphenomics.0164 (PMC11335093; doi:10.34133/plantphenomics.0164)

1. Supplementary Note 1- Summary of preset spectral index calculation formula

RGB refers to the RGB camera which contains three band: red(r), green(g) and blue(b). MS refers to the multi spectral camera which contains five band: blue, green, red, red edge(re) and near-infrared(nir).

| Index | Formula | Sensor |
| --- | --- | --- |
| BGI | $b/g$ | RGB/MS |
| BRI | $b/r$ | RGB/MS |
| CIVE | $r*0.441-g*0.811+b*0.385+18.78745$ | RGB/MS |
| EXG | $(g*2-r-b)/(r+g+b)$ | RGB/MS |
| EXR | $(r*1.4-g)/(r+g+b)$ | RGB/MS |
| EE | $EXG-EXR$ | RGB/MS |
| NDI | ${(g-r)}/{(g+r)}$ | RGB/MS |
| RGRI | $r/g$ | RGBMS |
| RGBVI | ${(g*g-b*r)}/{(g*g+b*r)}$ | RGB/MS |
| VARI | ${(g-r)}/{(g+r-b)}$ | RGB/MS |
| DVI | $nir-r$ | MS |
| GNDVI | ${(nir-g)}/{(nir+g)}$ | MS |
| MCARI1 | $1.2*(2.5*\left( nir-r \right)-1.3*(nir-g))$ | MS |
| MSAVI | $nir*2+1-\sqrt{{(nir*2+1)}^{2}-8*(nir-r)}$ | MS |
| MSR | ${({nir}/r-1)}/{(\sqrt{nir/r}+1)}$ | MS |
| NDRE | ${(nir-re)}/{(nir+re)}$ | MS |
| NDVI | ${(nir-r)}/{(nir+r)}$ | MS |
| RDVI | ${(nir-r)}/\sqrt{nir+r}$ | MS |
| OSAVI | $1.16*{(nir-r)}/{(nir+r+0.16)}$ | MS |
| RVI | ${nir}/r$ | MS |

1. Supplementary Note 2- Equations for regression modeling

Linear regression:

$$y=a*x+b$$

Exponential regression:

$$y=a*e^{b*x}$$

Quadratic regression:

$$y=a*x^{2}+b*x+c$$

Logarithmic regression

$$y=a*\ln x+b$$

Power regression:

$$y=a*x^{b}$$

1. Supplementary Note 3-Example Python Script for Image Feature based DL Extraction

import os

os.environ['TF_CPP_MIN_LOG_LEVEL'] = '2'

import sys

import argparse

import numpy as np

import os.path

# import required libraries, in this study model was trained by Keras

from keras.applications.vgg16 import preprocess_input

from keras.preprocessing import image

from keras.models import load_model

import h5py

# main

#img_path:the folder containing all the cropped plot images, specified by the IHUP automatically

parser.add_argument('img_path', type=str, help='Path to the image patch.')

parser.add_argument('feature_name')

args = parser.parse_args()

img_path = args.img_path

name = args.feature_name

# load model

model = load_model('./model1.h5')

rootdir = img_path

list = os.listdir(rootdir)

num = 0

for i in range(0,len(list)):

filename = list[i].split('.')[0]

path = os.path.join(rootdir,list[i])

img = image.load_img(path, target_size=(160, 160))

x = image.img_to_array(img)

x = np.expand_dims(x, axis=0)

LeafRollingScore = model.predict(x)*5#.reshape([,])

value = LeafRollingScore[0][0]

value = ("%.2f"%value)

#be sure to print the value in the following format

print('Marked_'+name+'_'+filename+'_'+str(value))

num=num+1

1. Supplementary Note 4-User Guide for IHUP

Step 0.

The original UAV photos have been generated into orthophotos through "image stitching", and crop height data, namely DEM or DSM, has been generated at the same time in this process. In the subsequent operation process, the orthophoto was recorded as DOM, which is represented by **[IMG]** in the software, and the crop height data was recorded as DSM, which was represented by **[DSM]** in the software.

Additional ROI data was needed to mark the location and label of each planting area. ROI data was a position range vector file manually sketched based on image, with suffix ‘.shp’. In the subsequent operation process, the cell file is recorded as ROI, and the software refers to **[ROI]**. All image data were required to name end in the format of ‘_YYYYMMDD’ which was convenient for batch processing.

Step1. Data Extraction

Store the IMG and DSM data in separate folders.

Click the button **[batching setting]** under the tab **[process]** to set the engineering parameters in the pop-up window, as shown in the following figure.


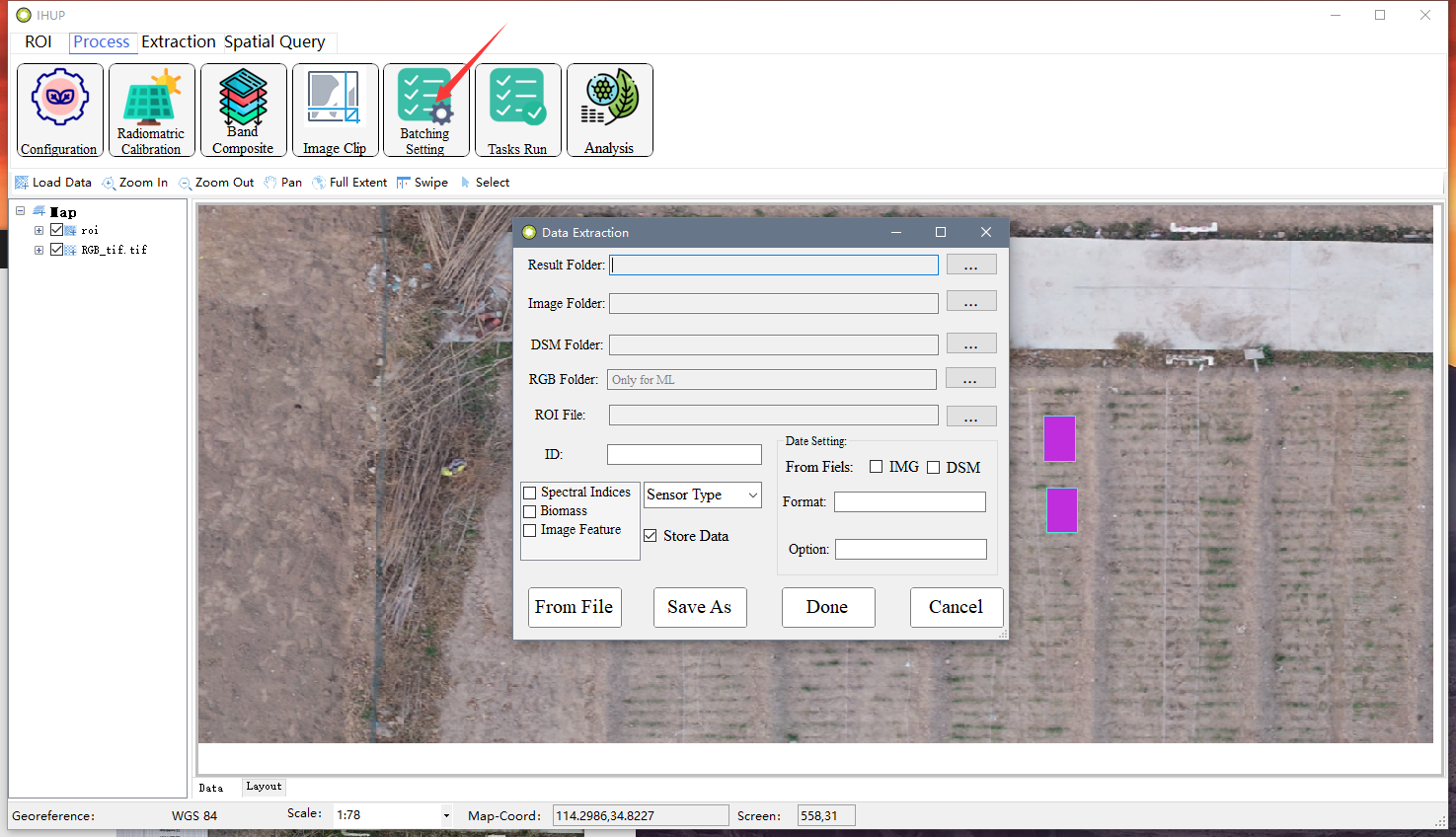


Parameters in the window could be added by selecting the button on the right.


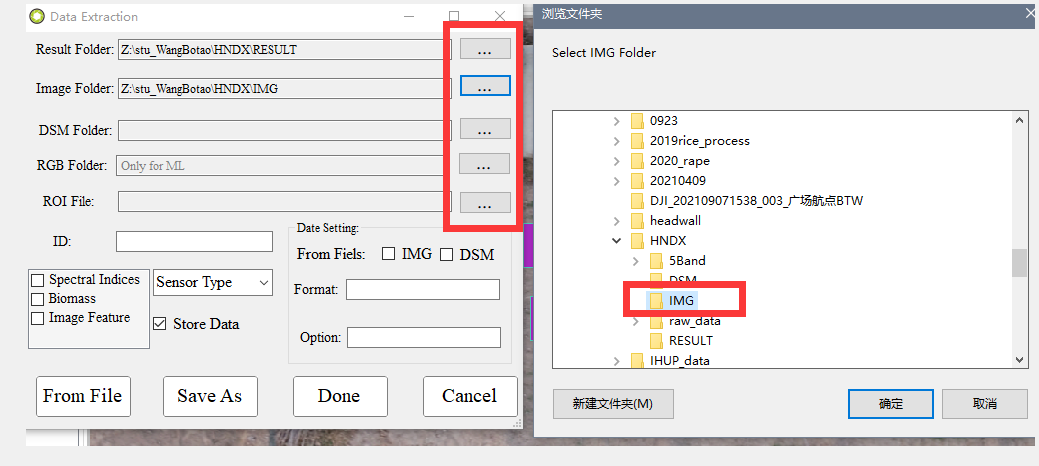


Click the button on the right side of the **[result folder]** to add the result output folder.

Click the button on the right side of the **[image folder]** to add a path containing IMG as shown in the above figure.

Click the button on the right side of the **[DSM folder]** to add a path containing DSM as shown in the following figure.


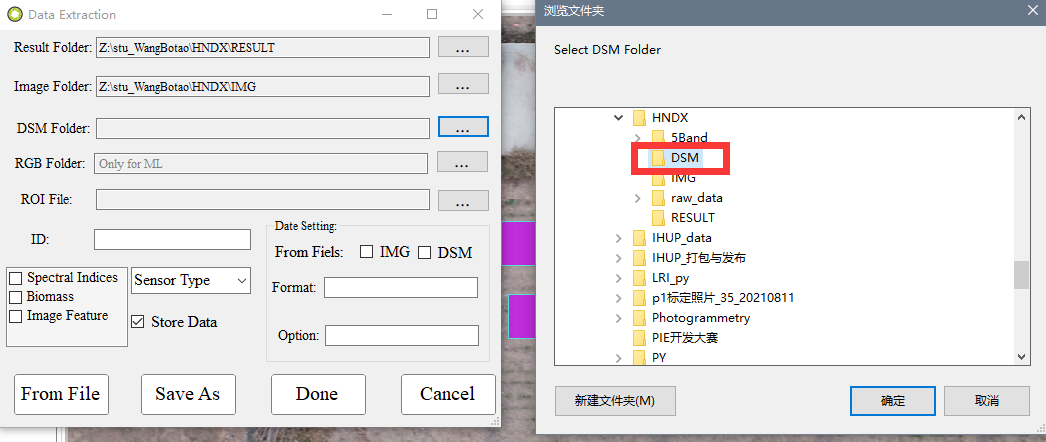


Click the button on the right side of the **[ROI folder]** and select ROI file in the pop-up window, as shown in the following figure


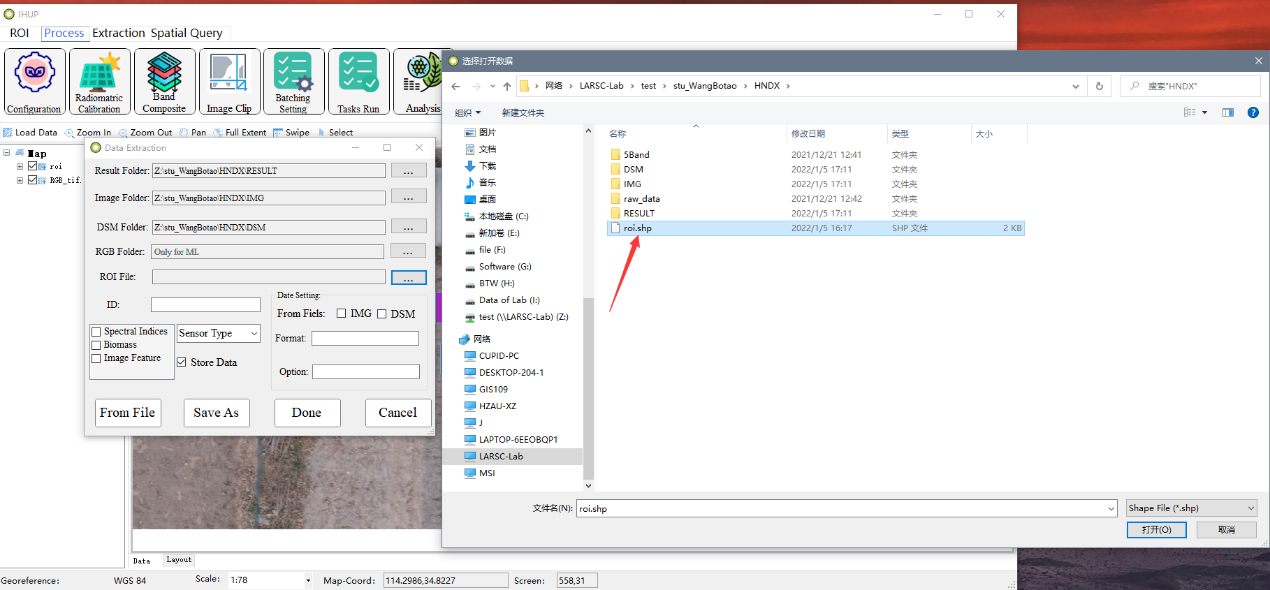


Set the task name for this processing, which could be specified as needed. Select the required calculated data. As shown in the following figure.


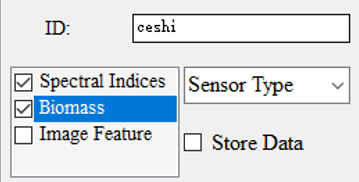


Check **[spectral indices]** to calculate the relevant spectral vegetation index according to the orthophoto.

Check **[biomass]** to calculate the crop height according to DSM and then estimate the dry weight and fresh weight according to the linear empirical relationship.

Check **[image feature]** to extract the specified image features according to the orthophoto and the configured depth learning model.

Select the sensor type, here we check [BGRNRE] for MS image data.

Do not check [Store Data] if there were no configured database.

Check [IMG] and the split strings between the file name and date stamp, ‘_’ were input.


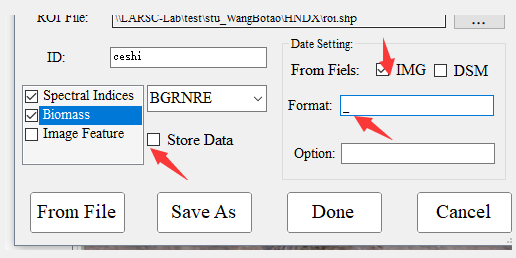


After all above settings were checked, click the [Done] to exit this window.

Click [tasks run] to open the window [batching], in which the task parameters set in the previous step will be displayed. After confirmation, click [run] to execute, as shown in the following figure:


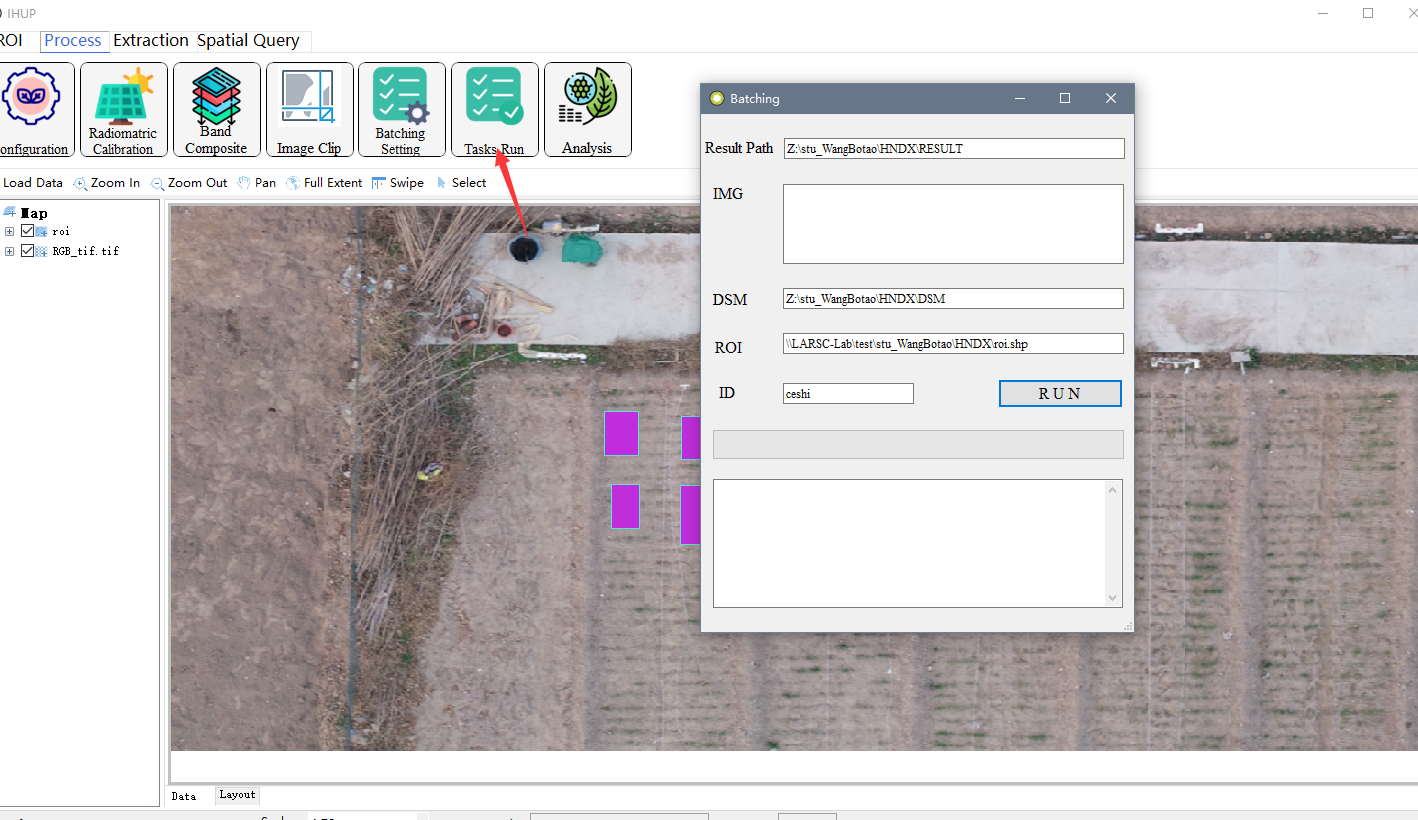


The lower interface will refresh the current task progress in real time. When the "mission completed" is prompted, the task is completed, as shown in the following figure


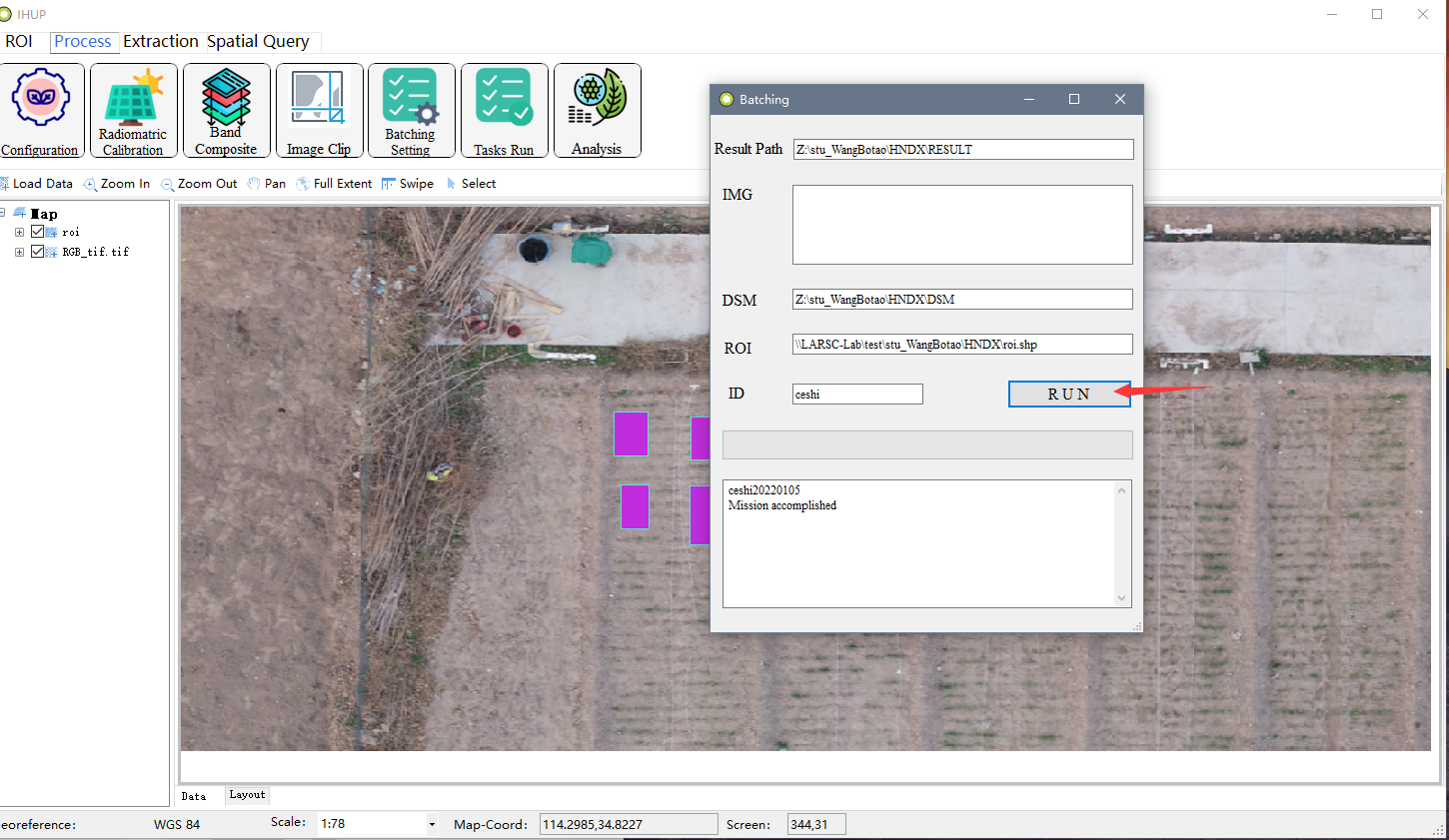


1. Supplementary Note 5-User Guide for Orthophoto and DSM Generation in Metashape

1.Data preparation:

The original data were the photos taken by the UAV, which could be either visible light (RGB) photos or multispectral (MS) photos.


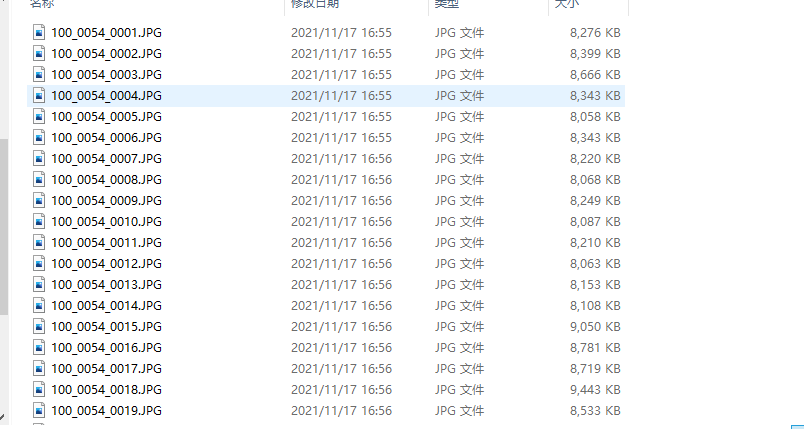


2.Creating workspace:

Click [New] under the bar “workspace” to create a new project. As shown in the figure below, “chunk” was right the current workspace.


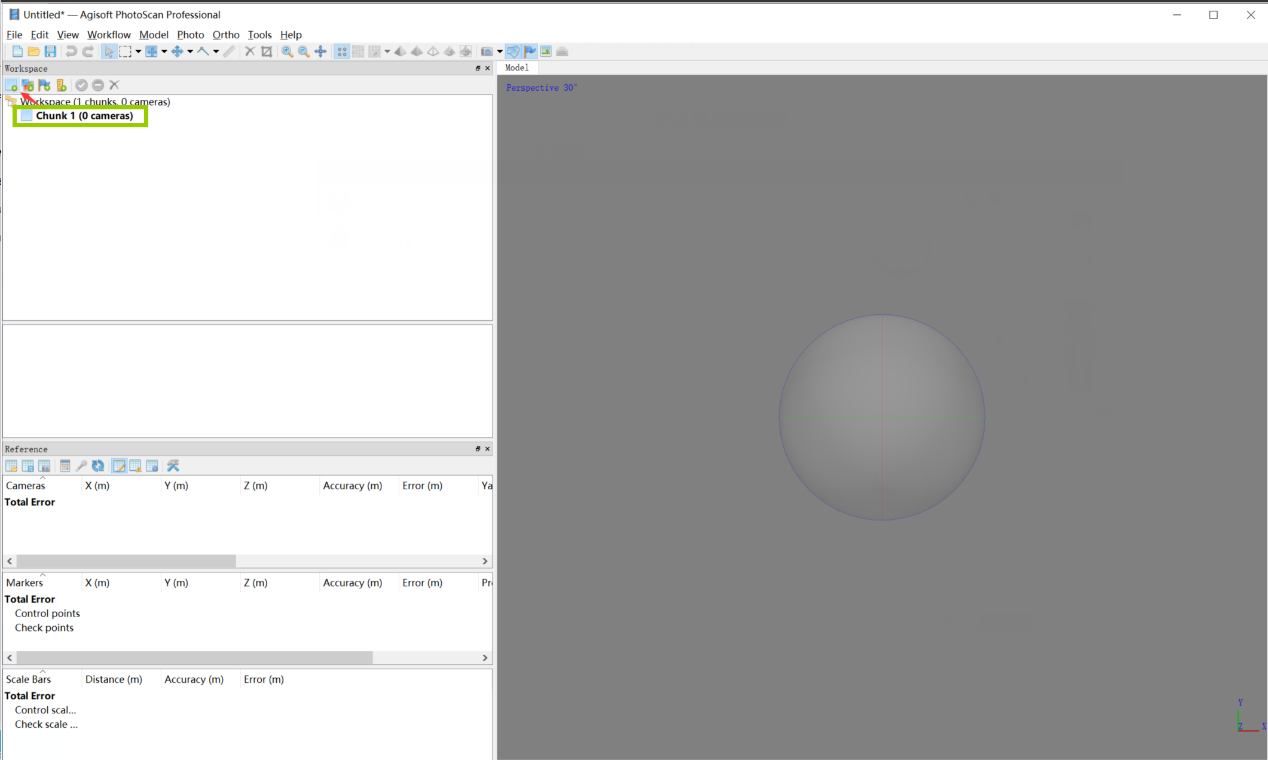


3.Adding photos

Click the second button from the left [add photos] to select photo in the pop-up menu.


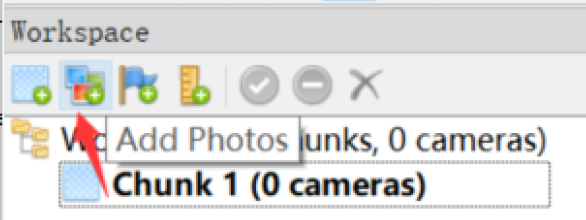


If target image was a multi-spectral image, the following dialog would pop up automatically after reading photos. Select [create multispectral camera], that was to merge five bands in to one image.


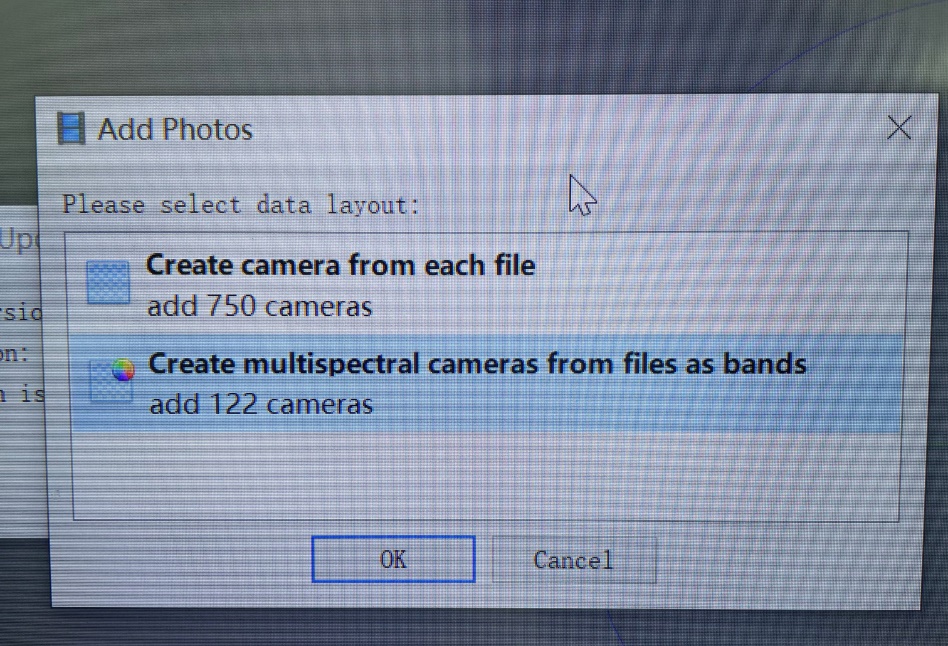


4.Orthophoto and DSM generation:

Following menu would pop up after click [Workflow].


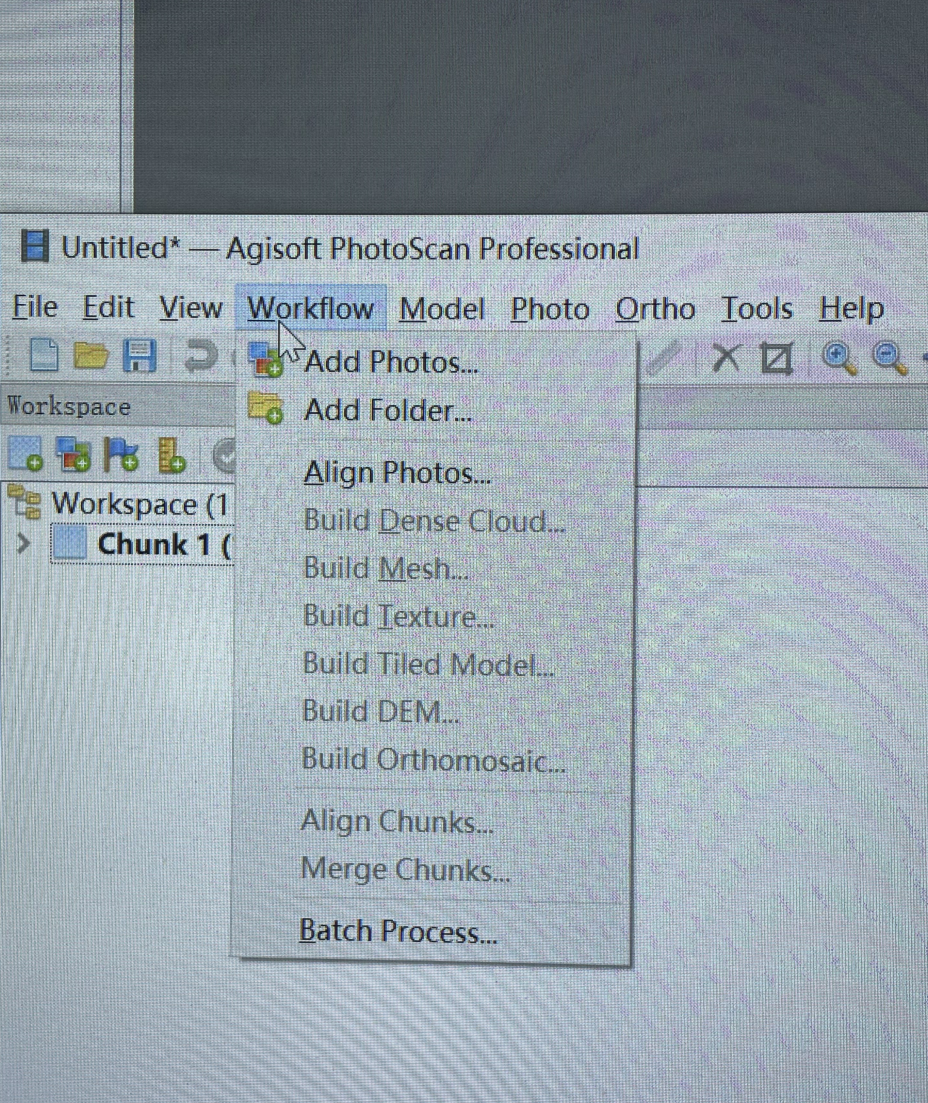


Click those buttons in turn to execute task: [Align Photos], [Build Dense Cloud], [Build Mesh], [Build Tiled Model], [Build DEM], [Build Orthomosaic].

The software would pop up windows similar to the following figure to determine the parameters, which could be executed according to the default settings. The accuracy of task could be changed as needed.


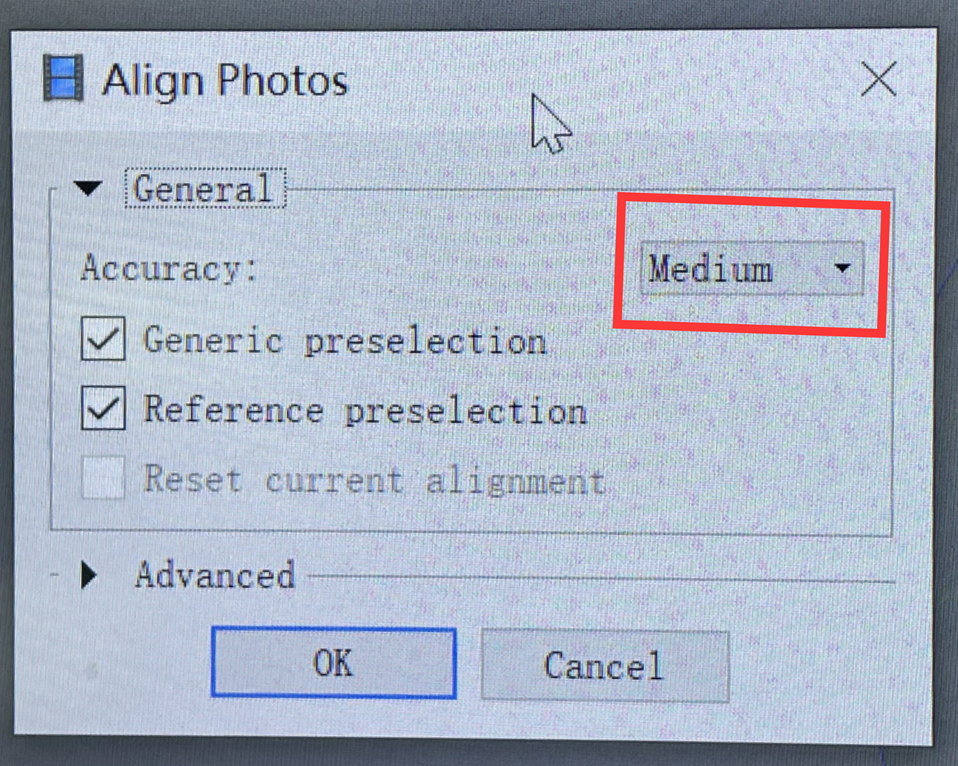


5.Data output

After generation, following data items would be displayed in the menu.


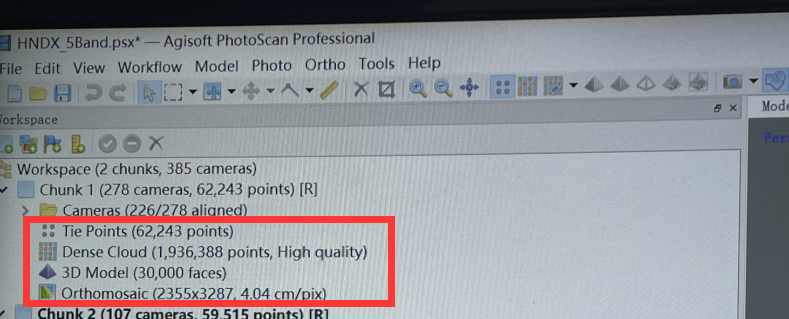


Right click [Orthomosaic], select and click [export orthomosaic] [export TIFF] in the pop-up menu.


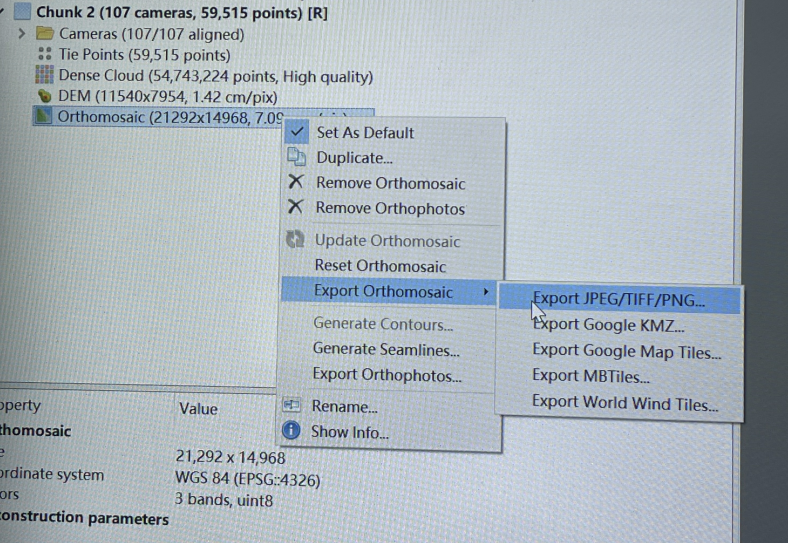


Specify the export path and set the parameters as default.


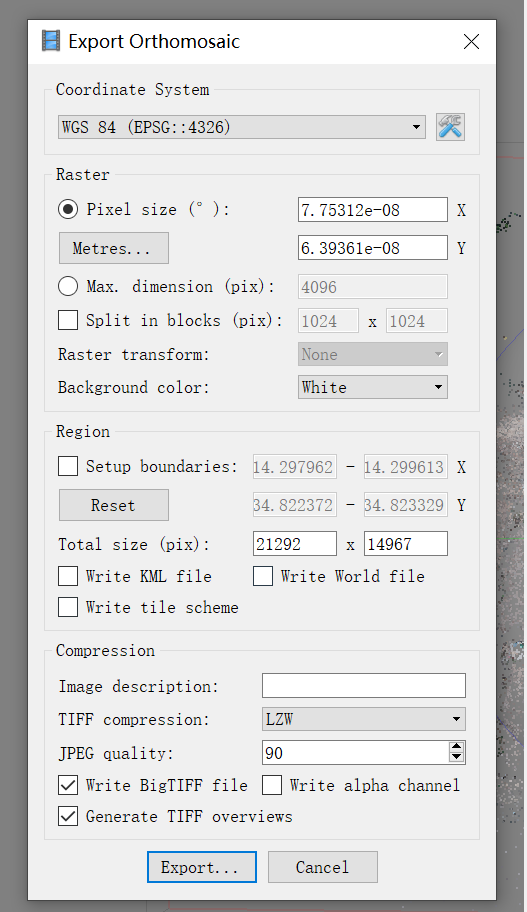

Supplement: Supplementary 1 — Notes S1 to S4 Movie S1 Tables S1 to S5 Guidelines for software testing [file plantphenomics.0164.f1.zip › SupplmentaryNote.docx]
